# Systemic inflammatory and metabolic disease progression persists despite prevention of myocarditis in *Isg15*-deficient Coxsackievirus B3 infection

**DOI:** 10.3389/fimmu.2026.1907453

**Published:** 2026-08-11

**Authors:** Sarah Ochs, Sandra Pinkert, Sophia Borowski, Lisa GM Huis in ‘t Veld, Nicolas Kelm, Ziya Kaya, Anne Hausen, Karin Klingel, Matthias M. Gaida, Antje Beling

**Affiliations:** 1 Charité – Universitätsmedizin Berlin, corporate member of Freie Universität Berlin and Humboldt-Universität zu Berlin, Institute of Biochemistry, Berlin, Germany; 2 Deutsches Zentrum für Herz-Kreislauf-Forschung, partner site Berlin, Berlin, Germany; 3 Department of Medicine I, Department of Gastroenterology and Hepatology, Faculty of Medicine and University Hospital Carl Gustav Carus, TU Dresden University of Technology, Dresden, Germany; 4 Kardiologie, Angiologie und Pneumologie, Medizinische Klinik für Innere Medizin III, Universitätsklinikum Heidelberg, Heidelberg, Germany; 5 Deutsches Zentrum für Herz-Kreislauf-Forschung (DZHK), Partner Site Heidelberg, Heidelberg, Germany; 6 Institute of Pathology, University Medical Center Mainz, Johannes-Gutenberg-Universität Mainz, Mainz, Germany; 7 Cardiopathology, Institute for Pathology and Neuropathology, University Hospital Tübingen, Tübingen, Germany; 8 TRON, Translational Oncology at the University Medical Center of the Johannes Gutenberg University Mainz, Mainz, Germany; 9 Research Center for Immunotherapy, University Medical Center Mainz, Johannes-Gutenberg-Universität Mainz, Mainz, Germany

**Keywords:** coxsackievirus B3, infection, inflammation, ISG15, microRNA targeting, viral myocarditis

## Abstract

**Introduction:**

Type I interferon responses are critical determinants of organ-specific antiviral control and metabolic adaptation during Coxsackievirus B3 (CVB3) infection. The interferon-stimulated gene ISG15 integrates antiviral defense with immunometabolic adaptation, and its deficiency aggravates viral myocarditis, systemic inflammation, metabolic wasting, and impaired cardiac performance. Previous studies further demonstrated impaired cardiac energetic adaptation in *Isg15*-deficient mice during CVB3 infection, raising the possibility that aggravated myocardial infection contributes to infection-associated wasting and systemic disease progression. We therefore investigated whether selective prevention of myocardial viral replication attenuates systemic inflammatory and metabolic deterioration during CVB3 infection in *Isg15*-deficient mice.

**Methods and results:**

*Isg15*-deficient mice were infected with control virus CVB3–39 or the cardiomyocyte-detargeted variant CVB3–1 containing miR-1 target sites. Cardiac detargeting profoundly reduced myocardial viral replication and prevented cardiomyocyte injury, inflammatory cell infiltration, and histological myocarditis. Selective suppression of myocardial viral replication did not substantially alter systemic viral dissemination or peripheral organ infection. Acute hepatitis and systemic cytokine responses developed similarly in CVB3-39– and CVB3-1–infected mice. Likewise, comparable reductions in cardiac output and ventricular filling parameters were observed in both infection groups during the acute phase of infection. Systemic metabolic alterations, including hypoglycemia, adipose tissue depletion, skeletal muscle wasting, and mortality, developed similarly with both viruses despite prevention of myocarditis with CVB3-1.

**Discussion:**

During CVB3 infection in *Isg15*-deficient mice, myocardial viral replication is essential for local myocarditis but does not substantially influence systemic inflammatory and metabolic disease progression. Prevention of myocarditis failed to attenuate inflammatory activation, metabolic wasting, early cardiac dysfunction, or mortality. These findings suggest that systemic disease manifestations primarily reflect defective antiviral and immunometabolic adaptation rather than heart-driven amplification of disease.

## Introduction

Innate immune signaling is a central determinant of the host immune response during enteroviral infection. Following recognition of viral nucleic acids by pattern-recognition receptors, type I interferons (IFN-I) induce broad antiviral transcriptional programs that restrict viral replication and coordinate tissue-specific defense mechanisms (1). In Coxsackievirus B3 (CVB3) infection, IFN-I signaling critically shapes disease progression at the organ level (2–7). Global deficiency of the type I interferon receptor (IFNAR) results in uncontrolled viral replication and increased mortality, highlighting the essential role of IFN-I in host protection (5). In cardiomyocytes, IFNAR signaling directly limits viral replication and accelerates viral clearance, thereby protecting against myocarditis progression (8). In parallel, IFN-I responses in hepatocytes are crucial for preventing hepatic necrosis and peripheral viral dissemination, establishing the liver as a major regulator of host-wide viral burden during CVB3 infection (4). Together, these studies indicate that interferon-mediated antiviral defense is uniformly organized across different tissues with programs that control local viral replication and inflammatory disease.

Among the most strongly induced interferon-stimulated genes (ISGs) during viral infection is ISG15, a ubiquitin-like modifier that is conjugated to target proteins through the enzymatic cascade of UBE1L, UBE2L6, and HERC5/6 in a process termed ISGylation (9). The CVB3 myocarditis model has been instrumental in establishing the biological relevance of ISG15 in antiviral host defense (10–13). CVB3 infection induces robust upregulation of ISG15 and the ISGylation machinery in cardiac tissue (10), while genetic deletion of ISG15 enhances viral replication and aggravates viral pathology *in vivo* (10, 11). Mechanistically, ISGylation directly modifies viral proteins, including the viral protease 2A, thereby interfering with viral protein processing and replication (11). In parallel, ISGylation stabilizes antiviral effector proteins such as members of the IFIT family and thereby amplifies intrinsic antiviral defense programs (10). Conversely, genetic inactivation of the de-ISGylating protease USP18 in mice enhances ISGylation and further suppresses viral burden in infected heart tissue (10), demonstrating that ISGylation functions as an active antiviral effector downstream of IFN-I signaling (14).

Beyond direct antiviral restriction, increasing evidence indicates that interferon signaling and ISG15 critically regulate cellular metabolism during infection (12, 15–17). IFN-I signaling influences glucose metabolism (12, 18, 19), mitochondrial function (12, 20), and bioenergetics, processes that are increasingly recognized as essential components of antiviral host defense (21). In CVB3 infection, ISG15 promotes mitochondrial respiration and bioenergetic adaptation in infected tissues (10, 12). Wild-type cardiomyocytes increase mitochondrial ATP production capacity during infection, whereas *Isg15-*deficient cells fail to mount this adaptive response (12). In parallel, ISGylation directly regulates multiple glycolytic enzymes and metabolic pathways, thereby modulating glycolytic flux and NAD+ regeneration (12, 18, 19). These findings position ISG15 as an immunometabolic regulator combining antiviral defense with metabolic adaptions during systemic infection.

Despite the established antiviral and metabolic functions of ISG15, the mechanistic relationship between aggravated myocarditis and systemic wasting during CVB3 infection remains unresolved. In *Isg15*-deficient mice, enhanced myocardial viral replication develops in parallel with systemic inflammation, progressive metabolic deterioration, severe wasting, and impaired cardiac performance (10–12). Previous work further demonstrated that *Isg15* deficiency compromises cardiac bioenergetic adaptation during infection, resulting in impaired mitochondrial ATP production capacity, altered glucose metabolism, and cardiac atrophy (4, 12). Because severe myocarditis and cardiac energetic dysfunction can alter circulatory homeostasis, increase inflammatory cytokine production (22, 23), and elevate organismal metabolic demand (12), the aggravated cardiac phenotype observed in *Isg15*-deficient mice could itself contribute to systemic metabolic deterioration. In particular, enhanced myocardial injury and energetic decompensation may amplify infection-associated wasting through inflammatory, hemodynamic, and metabolic mechanisms (22, 24). This raises the unresolved question of whether the pronounced body weight loss and muscle wasting observed in *Isg15*-deficient mice during CVB3 infection (10, 12) are secondary consequences of aggravated myocarditis or instead reflect a broader failure of antiviral and immunometabolic adaptation affecting multiple organs simultaneously.

Cardiomyocyte-specific viral detargeting using miR-1 target sequences provides a unique experimental strategy to address this question because it selectively suppresses myocardial viral replication while preserving systemic infection dynamics (25–27). Within the context of systemic *Isg15* deficiency, this model enables causal dissociation of aggravated myocarditis from the broader inflammatory and metabolic disease response and thereby allows direct testing of whether prevention of myocardial infection and cardiac injury is sufficient to attenuate systemic inflammation, metabolic wasting, and systemic disease progression during acute CVB3 infection.

## Materials and methods

### Animals

Male *Isg15*-deficient (*Isg15*^-/-^) mice (6–7 weeks of age) were housed at the Charité FEM breeding facility. The generation of this strain has been described previously (28). Mice were infected intraperitoneally with 10^5^ pfu/mouse of either CVB3–1 or CVB3–39 and maintained under standard housing conditions with ad libitum access to food and water. Body weight was recorded daily. Animals were euthanized on days 0, 3, or 7 post-infection by continuous inhalation of an isoflurane overdose (CP-Pharma, Burgdorf, Germany; approximately 15% vapor concentration generated in a sealed chamber using the drop method). Retrobulbar blood was collected immediately before euthanisation and blood glucose levels were measured with Accu-Chek glucometer (Roche Diabetes Care, Indianapolis, IN, USA). Prior to organ collection, transcardiac perfusion with PBS (Biochrom, Holliston, MA, USA) was performed. Harvested organs were weighed, and tissue samples were either processed for histology or snap-frozen in liquid nitrogen and stored at −80 °C. Pain management was provided via oral administration of 0.1 mg/mL tramadol hydrochloride (Grünenthal GmbH, Aachen, Germany) in the drinking water, as described previously (29). Animal welfare was monitored at minimum twice daily. Animals meeting predetermined termination criteria, defined as body weight loss exceeding 25%, or a sustained loss of more than 20% body weight persisting for longer than 24 hours, were humanely euthanized prior to the scheduled experimental endpoint. All animal experiments were approved by the local animal welfare authority in Berlin (LAGeSo; registration numbers G0119/20, T-CH0007-24, T0032/07) in accordance with the German Animal Welfare Act, the Animal Welfare Laboratory Regulations, and the European Parliament Directive 2010/63/EU on the protection of animals used for scientific purposes.

### Viruses

The construction of pMKS1-H3N-miR-1(3x)TS and pMKS1-H3N-39(3×)TS have been described previously (25, 30). Infectious viral particles of CVB3-miR-TS-1 (CVB3-1) and CVB3-miR-TS-39 (CVB3-39) were generated by transfecting these plasmids into HEK293T cells using polyethylenimine (PEI; Polysciences, Warrington, PA, USA). All resulting viral stocks were subsequently amplified through a single passage in HeLa cells.

### Echocardiography

To assess cardiac function, transthoracic echocardiography was performed in *Isg15*^-/-^ mice at baseline (day 0, one day prior to infection) and on day 3 post-infection, immediately prior to sacrifice, to assess cardiac function. Mice were anesthetized via continuous isoflurane inhalation (CP-Pharma, Burgdorf, Germany; 3–5% for induction, 1.5–2% for maintenance with an oxygen flow rate of 1 L/min), shaved, and positioned on a heated imaging platform with real-time monitoring of body temperature, respiration, electrocardiogram, and heart rate. Imaging was performed using a Vevo 3100 high-resolution ultrasound system (FUJIFILM VisualSonics, Toronto, ON, Canada) equipped with an MX400 transducer. The transducer was mounted on an adjustable holder and positioned on the thoracic wall using ultrasound gel. Throughout image acquisition, continuous physiological monitoring was maintained to ensure stable anesthesia and animal well-being throughout the procedure.

Standardized B-mode recordings were acquired in parasternal long-axis and mid-papillary short-axis views. M-mode measurements were obtained at the mid-papillary short-axis level to quantify left ventricular anterior (LVAW;d) and posterior (LVPW;d) wall thicknesses, left ventricular internal diameter in diastole (LVID;d), and myocardial mass (LVmass), the latter calculated using the standard geometric formula. Left ventricular end-diastolic and end-systolic volumes (LVEDV and LVESV), stroke volume (SV), cardiac output (CO), and ejection fraction (EF) were derived from endocardial border tracking.

Pulsed-wave Doppler signals of transmitral inflow and tissue Doppler signals of the septal mitral annulus were acquired from the apical four-chamber view. Pulsed-wave Doppler measurements yielded peak early (MV E) and atrial (MV A) diastolic transmitral flow velocities, as well as isovolumetric relaxation time (IVRT), isovolumetric contraction time (IVCT), and aortic ejection time (AET). Tissue Doppler imaging provided peak septal mitral annulus velocities during early diastole (MV E′), atrial contraction (MV A′), and systole (MV S′). Derived diastolic indices included the E/A and E/E′ ratios.

All recordings were digitally stored and analyzed offline using VevoLab software version 5.7.1 (FUJIFILM VisualSonics, Amsterdam, The Netherlands). All acquisition and analysis procedures were performed in accordance with current guidelines for small-animal echocardiography (31, 32).

### Serum parameters

Blood glucose levels were measured at baseline from tail vein and at endpoint via retrobulbar blood sampling using an Accu-Chek glucometer (Roche Diabetes Care, Basel, Switzerland). Whole blood was collected at endpoints via retrobulbar sampling and incubated for 30 min at RT. Serum was separated by centrifugation at 10,000 × g for 10 minutes and stored in aliquots at −80 °C until further analysis. Serum concentrations of bile acids, aspartate aminotransferase (AST), alanine aminotransferase (ALT), albumin, lipase, and lactate dehydrogenase (LDH) were determined by commercial analysis at ANTECH™ Diagnostics (Berlin, Germany). As elevated bile acid concentrations have been reported to confound metabolic and cardiovascular readouts, even in naïve mice (33), animals with bile acid levels exceeding twice the group mean were predefined as outliers and excluded from all subsequent analyses. Serum cytokine profiling was performed using the LEGENDplex™ Mouse Anti-Virus Response Panel (13-plex; BioLegend, San Diego, CA, USA) according to the manufacturer’s instructions. Samples were fixed with 2% ROTIHistofix (Carl Roth, Karlsruhe, Germany) in PBS for 30 minutes at RT and acquired on a BD FACSymphony™ A3 cell analyzer (BD Biosciences, Franklin Lakes, NJ, USA). Data analysis was carried out using the LEGENDplex™ Qognit cloud-based software (BioLegend). For high-sensitivity cardiac troponin T (hsTnT) quantification, serum samples were diluted 1:17.5 in 0.9% NaCl (B. Braun, Melsungen, Germany) and measured using an electrochemiluminescence immunoassay (ECLIA) on an Elecsys 2010 analyzer (Roche Diagnostics, Mannheim, Germany).

### Histology

Tissue samples designated for histological analysis were fixed in 4% ROTI™Histofix in PBS, subsequently embedded in paraffin, sectioned, and stained with hematoxylin and eosin (H&E). Stained slides were digitized using a NanoZoomer 2.0-HT scanner (Hamamatsu Photonics, Herrsching, Germany) and evaluated with the corresponding NDP.view2 software (Hamamatsu). Myocardial inflammation was quantified using an established myocarditis scoring system ranging from 0 to 4 (34). Pancreatic destruction was assessed on a scale of 0–100%, as described previously. Animals lacking complete pancreatic destruction at the endpoints (day 3 or 7 p.i.) were classified as non-responders and excluded from further analysis. Hepatic necrosis and inflammation were evaluated using an established histopathological scoring system (35). Hepatic glycogen was visualized in paraffin-embedded tissue sections by periodic acid-Schiff (PAS) staining. Sections were oxidized in 0.5% periodic acid solution for 5 minutes, followed by incubation with Schiff’s reagent for 15 minutes. Counterstaining was performed with Mayer’s hematoxylin. PAS-positive cells were scored semiquantitatively (10).

Skeletal muscle inflammation was assessed using two complementary scoring approaches. Inflammatory cell infiltration was graded on a scale from 0-3: 0 – no infiltration; 1 – mild infiltration; 2 – moderate infiltration; 3 – severe infiltration (36). Muscle fiber necrosis was additionally quantified using the Muscle Inflammation Score (MIS) (37), defined as follows: Grade 0 – no necrotic fibers; Grade 1 – up to 5 necrotic fibers per lesion; Grade 2–5 to 30 necrotic fibers; Grade 3 – more than 30 necrotic fibers; Grade 4 – diffuse extensive necrosis. When multiple lesions of identical grade were identified within a single muscle section, a score of 0.5 points was added accordingly. In muscle sections, macrophages were detected using an F4/80 antibody (BM8, 14-4801-85, eBioscience™, Invitrogen, Waltham, Massachusetts, USA), and the number of F4/80-positive cells was digitally quantified in cells/mm^2^. All histopathological scoring was performed by blinded pathologists.

### Cell culture experiments

HeLa cells (ATCC CCL-2) were maintained in minimum essential medium (MEM; Thermo Fisher Scientific, Carlsbad, CA, USA) supplemented with 5% fetal calf serum (FCS; Sigma-Aldrich, Burlington, MA, USA), 1% penicillin/streptomycin, 1% non-essential amino acids (NEAA), and 20 mM HEPES (all Gibco/Life Technologies, Carlsbad, CA, USA). HEK293T cells (ATCC) were cultured in Dulbecco’s modified Eagle’s medium (DMEM; Gibco/Life Technologies, Carlsbad, CA, USA) supplemented with 10% FCS, 1% penicillin/streptomycin, and 1 mM sodium pyruvate (Gibco/Life Technologies, Carlsbad, CA, USA). Both cell lines were maintained at 37 °C in a humidified atmosphere containing 5% CO_2_ and passaged three times per week.

### Quantification of virus titer

Viral titers from cell lysates, and mouse tissue homogenates were determined by plaque assay on confluent HeLa cell monolayers as previously described (29). Prior to assay, cells were lysed by three freeze–thaw cycles, and organs were homogenized using a FastPrep-24 Classic instrument (MP Biomedicals, Santa Ana, CA, USA). Serial 10-fold dilutions of all samples were prepared, and all plaque assays were performed in duplicate. Confluent HeLa cells were inoculated with each dilution and incubated for 30 minutes at 37 °C. Following inoculation, the supernatant was carefully removed, and cells were overlaid with Eagle’s agar, composed of MEM supplemented with 1% penicillin/streptomycin, 3.2 g/L NaHCO_3_, 9% FCS (Sigma-Aldrich, St. Louis, MO, USA), and 0.7% BD Difco Agar Noble (Thermo Fisher Scientific, Waltham, MA, USA). After 2 days of incubation, cells were stained with MTT (3-(4,5-dimethylthiazol-2-yl)-2,5-diphenyltetrazolium bromide; Sigma-Aldrich) for up to 4 hours, after which plaques were counted.

### RNA-isolation and quantitative reverse transcription PCR (RT-qPCR)

RNA was extracted using TRIzol reagent (Thermo Fisher Scientific, Waltham, MA, USA), snap-frozen, and stored at −80 °C until further analysis. RT-qPCR was performed as previously described (38). Gene expression of *Ifit3, Ifn-b, Il-1b, Tnf-a, Ccl2* and *Cxcl10* was quantified using TaqMan gene expression assays (Thermo Fisher Scientific, Waltham, MA, USA). Custom primers and probes were used for the detection of murine *Hprt* and CVB3 genomic RNA, with sequences as follows: murine *Hprt* (forward primer: 5′-ATC ATT ATG CCG AGG ATT TGG AA-3′; reverse primer: 5′-TTG AGC ACA CAG AGG GCC A-3′; probe: 5′FAM-TGG ACA GGA CTG AAA GAC TTG CTC GAG ATG-3′ TAMRA) and CVB3 (forward primer: 5′-CCC TGA ATG CGG CTA ATC C-3′; reverse primer: 5′-ATT GTC ACC ATA AGC AGC CA-3′; probe: 5′-FAM-TGC AGC GGA ACC G-MGB3′). Relative quantification was performed using the ΔC(t) method, with *Hprt* serving as the endogenous reference gene.

### Immune cell isolation and flow cytometry

Heart tissue (20 mg) was minced into small pieces in ice-cold RPMI (Gibco/Life Technologies, Carlsbad, CA, USA) supplemented with 2% FCS, 30 mM HEPES, and 1% penicillin/streptomycin. Collagenase type II (1 mg/mL; Worthington Biochemical Corporation, Lakewood, NJ, USA) and DNase I (0.15 mg/mL; Sigma-Aldrich) were added, and tissue was digested under continuous shaking for 30 minutes at 37 °C. Enzymatic digestion was terminated by addition of 10 mM EDTA. Cell suspensions were passed through a 70-µm cell strainer and centrifuged at 310 × g for 10 minutes at 4 °C. Red blood cell lysis was performed using ammonium chloride-based lysis buffer (155 mM NH_4_Cl, 10 mM KHCO_3_, 0.1 mM EDTA) for 3 minutes at room temperature, after which lysis was stopped by addition of FACS buffer (PBS containing 2% FCS and 2 mM EDTA). Cells were centrifuged and resuspended in FACS buffer.

Spleens were mechanically dissociated by passage through a 70-µm cell strainer, followed by red blood cell lysis as described above. Splenocytes were used exclusively for single-stain compensation controls.

Liver tissue was minced and digested in RPMI supplemented with 0.1% BSA (AppliChem), 1% penicillin/streptomycin, and 0.2% collagenase type IV (Worthington Biochemical Corporation, Lakewood, NJ, USA) for 20 minutes at 37 °C. DNase I was subsequently added to a final concentration of 0.9 mg/mL, and digestion was continued for an additional 20 minutes under identical conditions. Digestion was terminated using HBSS (Thermo Fisher Scientific, Waltham, MA, USA) containing 0.1% BSA and 2 mM EDTA. Cell suspensions were filtered through a 70-µm strainer and washed twice by low-speed centrifugation at 55 × g for 1 minute at 4 °C to remove debris, with the pellet discarded each time. A final centrifugation at 500 × g for 10 minutes at 4 °C was performed, and the resulting pellet was resuspended in 6 mL of 30% Nycodenz solution (Serumwerk Bernburg, Bernburg, Germany). Four milliliters of HBSS containing 0.1% BSA was added, and an additional 6 mL of the same buffer was carefully layered on top to establish two distinct phases. Density gradient centrifugation was carried out at 1,400 × g for 22 minutes at 4 °C with slow acceleration and no brake. Leukocytes were collected from the interphase, washed, and resuspended in FACS buffer. Cell concentration was determined using a Neubauer hemocytometer. All cell suspensions were kept on ice until staining.

Isolated immune cells from heart, spleen, and liver were first incubated with Fc-blocking reagent (1:50 dilution; Miltenyi Biotec) for 20 minutes at 4 °C. Cells were subsequently stained with antibody cocktails for 20 minutes at 4 °C in the dark. All antibodies are listed in **Supplementary Table 1** and were obtained from BD Biosciences, BioLegend (San Diego, CA, USA), and Life Technologies. Following a wash step, viability staining was performed using Fixable Viability Dye eFluor™ 780 (1:1000 dilution; eBioscience, San Diego, CA, USA) for 30 minutes at 4 °C in the dark. Cells were then washed and fixed in 2% ROTI™Histofix (Carl Roth, Karlsruhe, Germany) for 30 minutes at room temperature, followed by a final wash and resuspension in FACS buffer. For absolute immune cell quantification, 123count eBeads (Thermo Fisher Scientific, Waltham, MA, USA) were added to heart samples prior to acquisition. Samples were acquired on a BD FACSymphony™ A3 cell analyzer (BD Biosciences, Franklin Lakes, NJ, USA), and data were analyzed using FlowJo software version 10.9.0 (BD Biosciences). Gating strategies are provided in **Supplementary Figures 3–8**.

## Statistics

**Figure 1:**
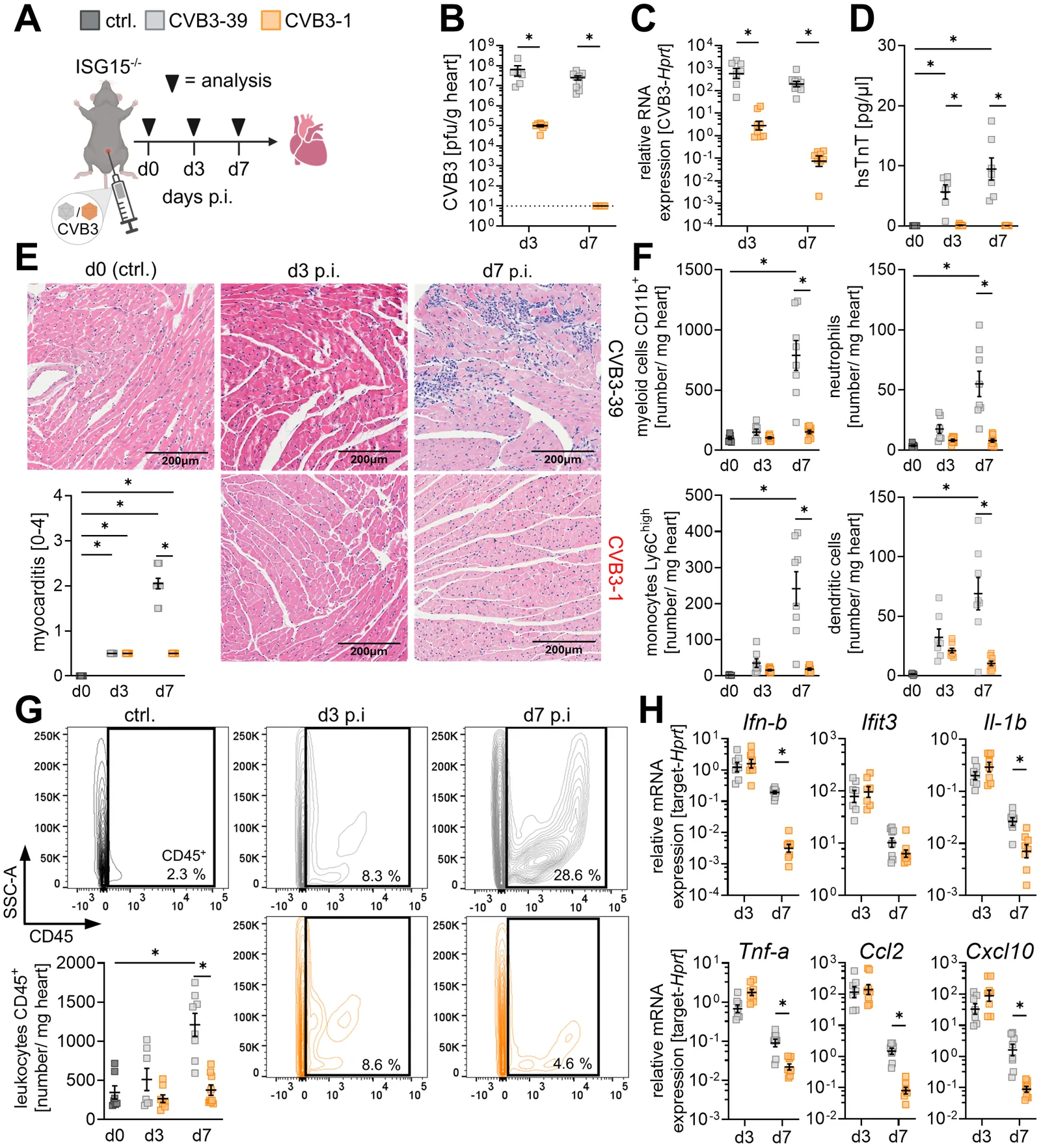
Cardiomyocyte detargeting prevents myocardial viral replication and myocarditis in *Isg15*-deficient mice. **(A)** Experimental design. Six- to seven-week-old *Isg15*^-/-^ mice were infected intraperitoneally with 10^5^ PFU/mouse of replication-competent CVB3–39 or the cardiomyocyte-detargeted variant CVB3–1 and the heart was analyzed at day 3 (acute phase) and day 7 (subacute phase). Age-matched uninfected mice served as baseline controls (day 0). Group sizes: d0 (N = 8), d3 CVB3-39 (N = 9), d3 CVB3-1 (N = 9), d7 CVB3-39 (N = 10), d7 CVB3-1 (N = 10). (B/C) Viral burden was quantified in the heart. Infectious viral titers were determined by plaque assay **(B)**, and viral genome levels were quantified by RT-qPCR **(C)**. **(D)** Serum troponin T levels were measured using an electrochemiluminescence immunoassay (ECLIA). **(E)** Representative H&E-stained heart sections at the indicated time points. Myocarditis was scored histologically. Scale bar, 200 µm. **(F, G)** Cardiac immune cell populations were analyzed by flow cytometry. **(F)** Quantification of CD45^+^CD11b^+^ myeloid cells, CD45^+^CD11b^+^ Ly6G^+^ neutrophils, CD45^+^CD11b^+^ Ly6C high inflammatory monocytes, and CD45^+^CD11b^+^ CD11c^+^ dendritic cells (DC). **(G)** Representative contour plots of CD45^+^ leukocytes for both viral strains with corresponding quantification. **(H)** mRNA expression of interferon/interferon-stimulated genes (IFN/ISG), cytokines, and chemokines in cardiac tissue measured by RT-qPCR. Data are shown as mean ± SEM. Outliers were detected using the ROUT (Q = 1%) method. Two-way ANOVA with Sidak’s *post hoc* test was used for datasets with two independent factors (time and virus strain) **(B–D, F–H)**. One-way ANOVA with Dunnett’s *post hoc* test compared post-infection time points to baseline **(D, F, G)**. Ordinal data were evaluated using a Mann–Whitney U test **(E)**. Statistical significance was defined as p ≤ 0.05 (*).

**Figure 2:**
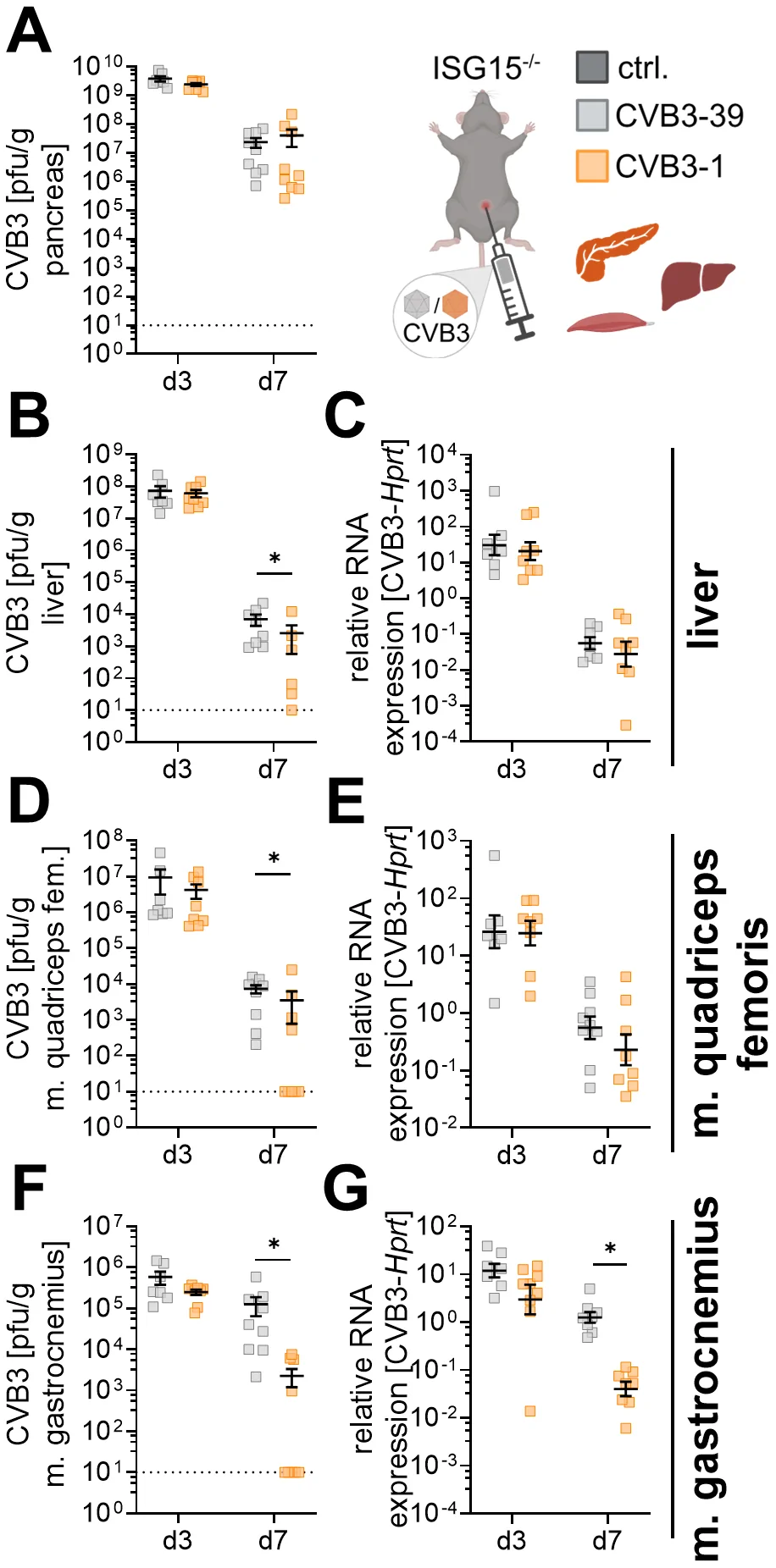
Peripheral viral replication remains largely preserved following cardiomyocyte detargeting. Six- to seven-week-old *Isg15*^-/-^ mice were infected intraperitoneally with 10^5^ PFU/mouse of CVB3–39 or CVB3–1 and pancreas, liver and muscle were analyzed at day 3 (acute phase) and day 7 (subacute phase). Age-matched uninfected mice served as baseline controls (day 0). Group sizes: d0 (N = 8), d3 CVB3-39 (N = 9), d3 CVB3-1 (N = 9), d7 CVB3-39 (N = 10), d7 CVB3-1 (N = 10). Viral burden was quantified in the pancreas **(A)**, liver **(B, C)**, musculus quadriceps femoris **(D, E)** and musculus gastrocnemius **(F, G)**. Infectious viral titers were determined by plaque assay **(A, B, D, F)**, and viral genome levels were quantified by RT-qPCR **(C, E, G)**. Data are shown as mean ± SEM. Outliers were detected using the ROUT (Q = 1%) method. Two-way ANOVA with Sidak’s *post hoc* test was used for datasets with two independent factors (time and virus strain) **(A–G)**. Statistical significance was defined as p ≤ 0.05 and is indicated by a single asterisk (*).

**Figure 3:**
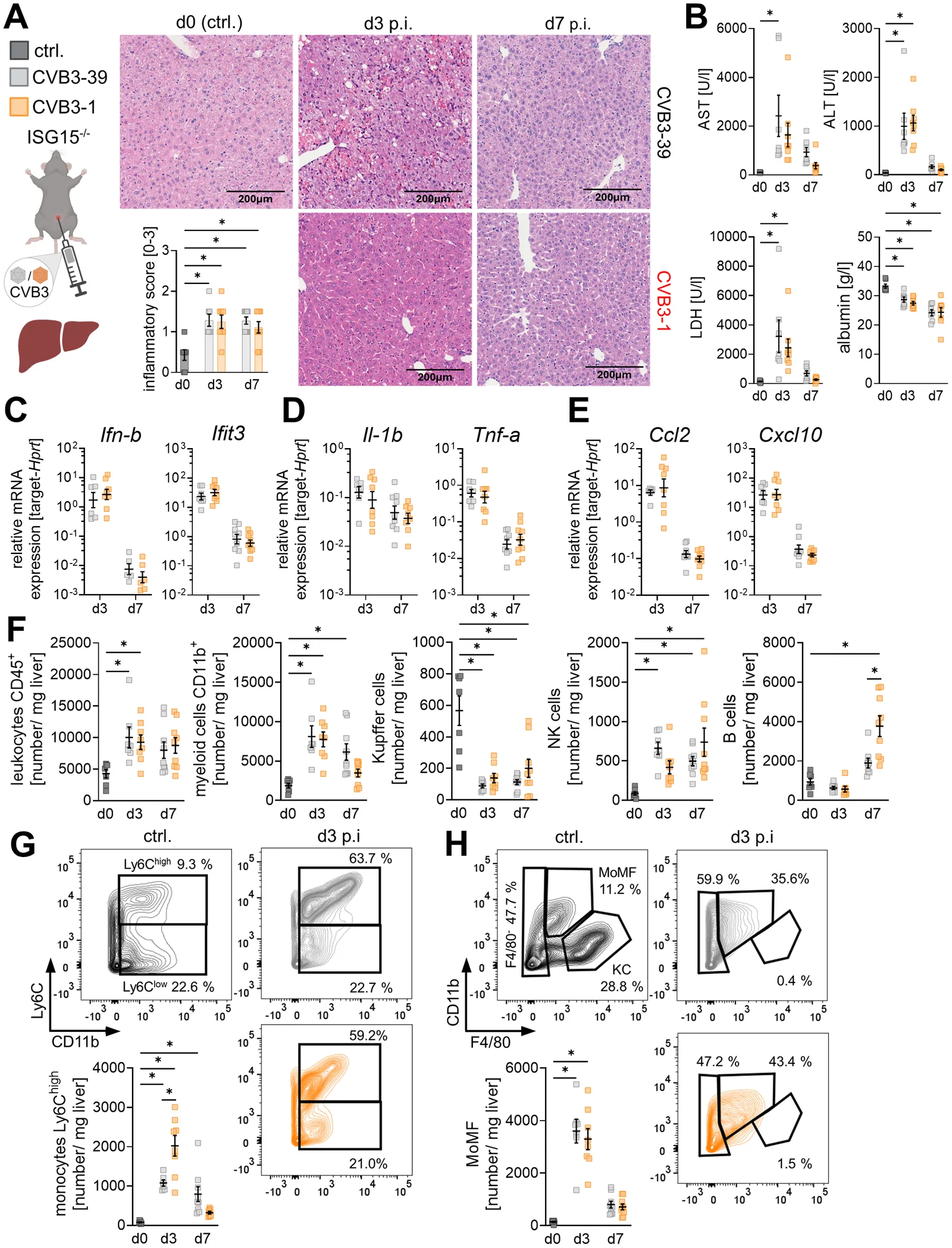
Acute hepatitis develops independently of myocardial viral replication. Six- to seven-week-old *Isg15*^-/-^ mice were infected intraperitoneally with 10^5^ PFU/mouse of CVB3–39 or CVB3–1 and liver was analyzed at day 3 (acute phase) and day 7 (subacute phase). Age-matched uninfected mice served as baseline controls (day 0). Group sizes: d0 (N = 8), d3 CVB3-39 (N = 9), d3 CVB3-1 (N = 9), d7 CVB3-39 (N = 10), d7 CVB3-1 (N = 10). **(A)** Representative H&E–stained liver sections at the indicated time points. Scale bar, 200 µm. Liver inflammation and necrosis were scored according to the inflammatory scoring system described by (35). **(B)** Serum aspartate aminotransferase (AST), alanine aminotransferase (ALT), lactate dehydrogenase (LDH) and albumin levels. **(C–E)** mRNA expression of interferon/interferon-stimulated genes (IFN/ISG) **(C)**, cytokines **(D)**, and chemokines **(E)** in liver tissue measured by RT-qPCR. **(G–I)** Immune cell populations were analyzed by flow cytometry and cell numbers per milligram of liver tissue are shown for the following populations: CD45^+^ leukocytes, CD45^+^ CD11b^+^ myeloid cells, CD45^+ -^CD11b^intermediate^F4/80^high^ Kupffer cells (KC). Additionally, following lymphoid populations are shown: CD45^+ -^NK1.1^+^ NK cells, CD45^+^CD19^+^B220^+^ B-cells **(F)**. (G/H) Representative contour plots are shown for uninfected mice and at day 3 post infection for both viral strains. **(G)** CD45^+^ CD11b^+^ Ly6C^high^ inflammatory and Ly6C^low^ patrolling monocytes and quantification of Ly6C^high^ cells per mg liver tissue. **(H)** CD45^+^ CD11b^high^F4/80^intermediate^ monocyte-derived macrophages (MoMF) together with KC and quantification of MoMF per mg liver tissue. Data are shown as mean ± SEM. Outliers were detected using the ROUT (Q = 1%) method. Two-way ANOVA with Sidak’s *post hoc* test was used for datasets with two independent factors (time and virus strain) **(B-H)**. One-way ANOVA with Dunnett’s *post hoc* test compared post-infection time points to baseline (B/F-H). Ordinal data were evaluated using a Mann–Whitney U test **(A)**. Statistical significance was defined as p ≤ 0.05 (*).

**Figure 4:**
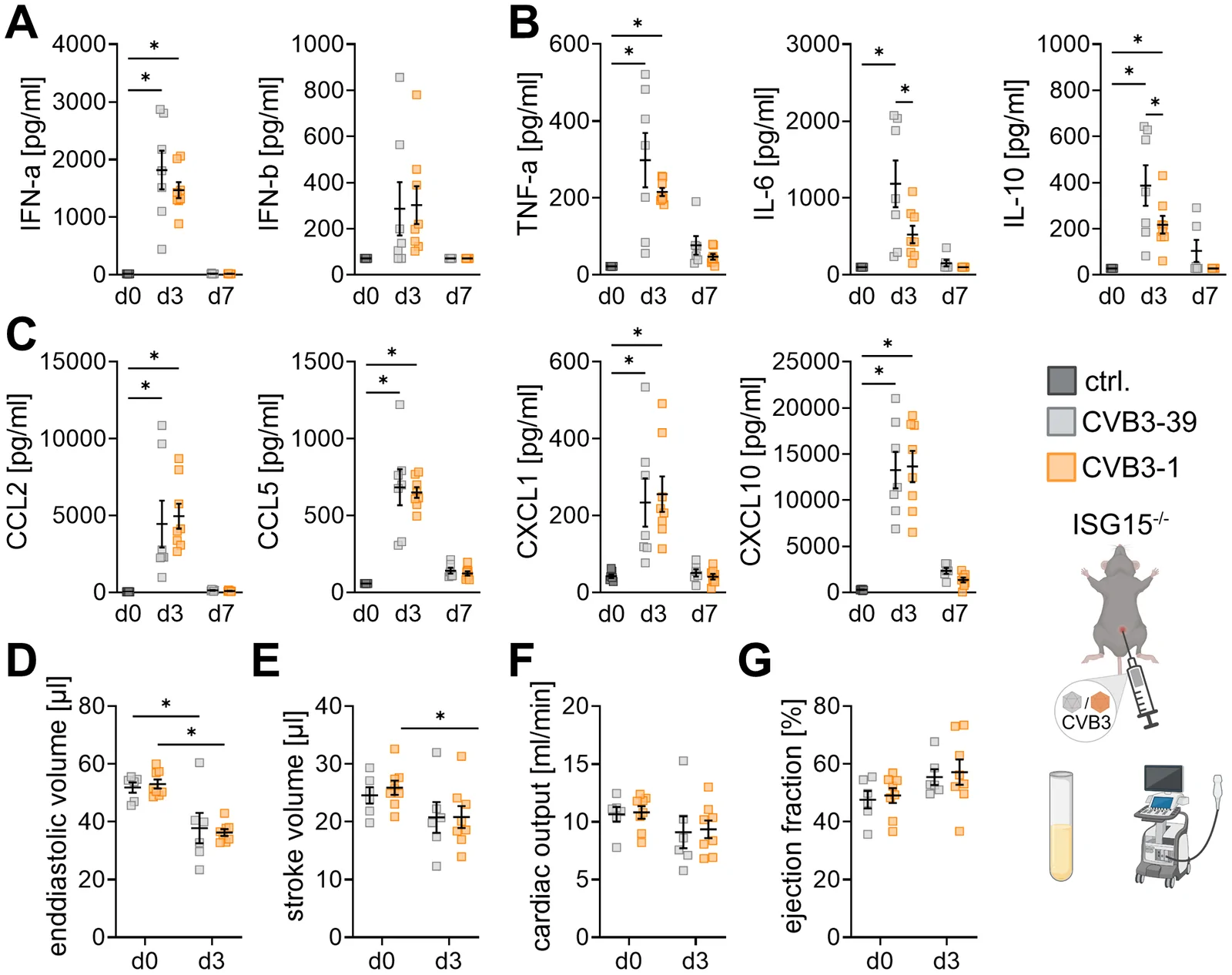
Systemic inflammatory activation and early cardiac dysfunction occur independently of myocardial viral replication. Six- to seven-week-old *Isg15*^-/-^ mice were infected intraperitoneally with 10^5^ PFU/mouse of CVB3–39 or CVB3–1 and serum was analyzed at day 3 (acute phase) and day 7 (subacute phase). Age-matched uninfected mice served as baseline controls (day 0). Group sizes: d0 (N = 8), d3 CVB3-39 (N = 9), d3 CVB3-1 (N = 9), d7 CVB3-39 (N = 10), d7 CVB3-1 (N = 10). Serum levels of interferons: IFN-a, IFN-b **(A)**; cytokines: TNFa-, IL-6, IL-10 **(B)**; and chemokines: CCL2, CCL5, CXCL1, CXCL10 **(C)** were measured using a LEGENDplex bead-based multiplex immunoassay. Transthoracic echocardiography was performed one day prior to infection (baseline) and at day 3 (acute phase) immediately before sacrifice. Group sizes were as follows: baseline: CVB3–39 N = 6, CVB3–1 N = 8, d3: CVB3–39 N = 6 and CVB3–1 N = 8. Left ventricular systolic function and volumes were assessed as follows: left ventricular end-diastolic volume (LVEDV) **(D)**, stroke volume **(E)**, cardiac output **(F)**, and ejection fraction **(G)**. Data are shown as mean ± SEM. Two-way ANOVA with Sidak’s *post hoc* test was used for datasets with two independent factors (time and virus strain). One-way ANOVA with Dunnett’s *post hoc* test compared post-infection time points to baseline **(A–C)**. Changes in echocardiographic parameters within the same animal over time were analyzed using paired t-tests **(D–G)**. Statistical significance was defined as p ≤ 0.05 (*).

**Figure 5:**
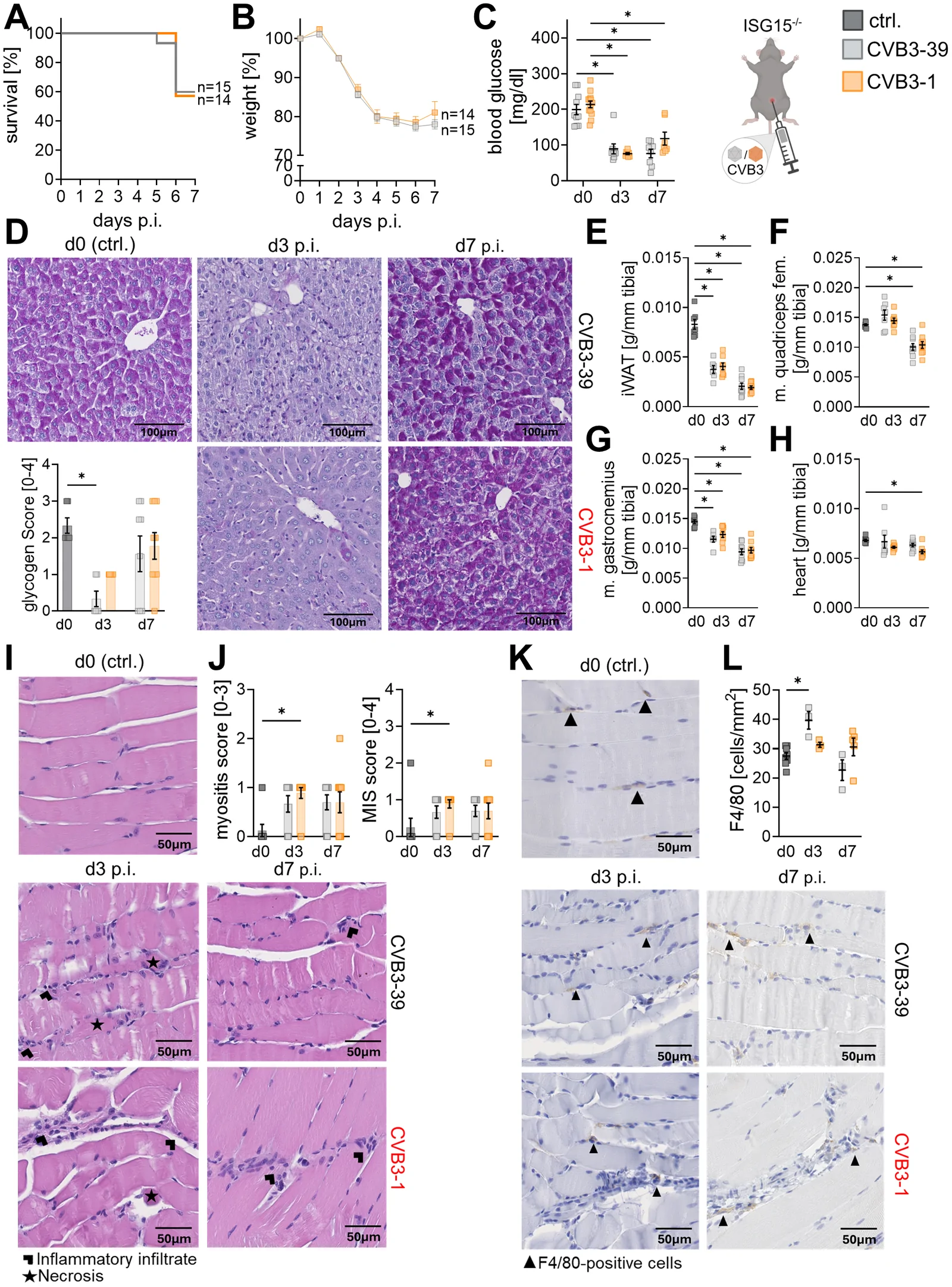
Systemic metabolic deterioration persists despite suppression of myocardial viral replication. Six- to seven-week-old *Isg15*^-/-^ mice were infected intraperitoneally with 10^5^ PFU/mouse of CVB3–39 or CVB3–1 and the mice were analyzed at day 3 (acute phase) and day 7 (subacute phase). Age-matched uninfected mice served as baseline controls (day 0). Group sizes: d0 (N = 8), d3 CVB3-39 (N = 9), d3 CVB3-1 (N = 9), d7 CVB3-39 (N = 10), d7 CVB3-1 (N = 10) if not indicated otherwise. **(A, B)** Kaplan-Meier survival curves **(A)** and body weight change over the course of infection **(B)** for CVB3-39 (N = 15) and CVB3-1 (N = 14) infected mice. **(C)** Blood glucose levels were measured with a glucometer. **(D)** Glycogen content visualized in liver tissue section with periodic acid Schiff reaction. Representative images are shown and glycogen content was scored semiquantitative from 0 to 4 based on histological analysis described before (10). **(E–H)** Organs were weighed and the organ weight was normalized to the tibia length for **(E)** inguinal white adipose tissue (iWAT), **(F)** musculus quadriceps femoris (m. quadriceps fem.), **(G)** musculus gastrocnemius (m. gastrocnemius) and **(H)** heart. **(I)** Representative images from H&E-stained skeletal muscle sections at the indicated time points. Inflammatory infiltrates are indicated by arrows and necrotic areas by asterisks. Scale bar, 50 µm. **(J)** Histological quantification of myositis score and muscle inflammatory score (MIS). **(K)** Immunohistochemistry staining for F4/80^+^ macrophages in the histologic sections of skeletal muscle was performed. Representative images are shown. **(L)** Quantification of the F4/80 staining. Data are shown as mean ± SEM. Outliers were detected using the ROUT (Q = 1%) method. Two-way ANOVA with Sidak’s *post hoc* test was used for datasets with two independent factors (time and virus strain) **(C, E–H, L)**. One-way ANOVA with Dunnett’s *post hoc* test compared post-infection time points to baseline **(E-H, L)**. Ordinal data were evaluated using a Mann–Whitney U test **(D, J)**. Survival curves were analyzed using log-rank (Mantel–Cox) test **(A)** and mixed effect model (REML) was used for repeated measurements **(B)**. Statistical significance was defined as p ≤ 0.05 and is indicated by a single asterisk (*).

Statistical analyses were performed using GraphPad Prism version 10.5.0 for Windows (GraphPad Software, La Jolla, CA, USA). For data shown in **Figures 1B–D, F–H**, **2A–G**, **3A–H**, **4A–C**, **5C, E–H, L**; and **Supplementary Figures 1A–D**, **2A–C**, outliers were identified using the ROUT method (Q = 1%) and excluded from analysis. Data are presented as individual values with mean ± SEM unless stated otherwise. Normality of data distribution was assessed using the D’Agostino–Pearson test. Viral titers are displayed on a log10-scaled y-axis and were log10-transformed prior to statistical analysis. Relative gene expression was calculated using the 2^−ΔCt^ method and log10-transformed for statistical testing and visualization. For echocardiographic analyses, comparisons between virus groups at individual time points and for comparisons of the same virus strain across different days post-infection, unpaired t-tests or Mann–Whitney U tests, when normality was not met, was applied (**Figures 4D–G**; **Supplementary Figure 7I–K**; **Table 1**). Changes in echocardiographic parameters within the same animal over time were analyzed using paired t-tests (**Tables 2**, **3**).

**Table 1:** Comparative echocardiographic analysis of CVB3-1– and CVB3-39–infected mice.

% changes to baseline	Day 3		Day 3: CVB3–1 vs. CVB3.39
	CVB3-1	CVB3-39	
LV Performance			
Heart rate [%]	101.2 ± 6.0	108.9 ± 4.7	ns
LVEDV [%]	73.1 ± 9.8	68.9 ± 3.4	ns
LVESV [%]	64.4 ± 12.0	58.7 ± 7.2	ns
Stroke volume [%]	85.7 ± 10.4	80.6 ± 6.6	ns
Cardiac output [%]	87.4 ± 13.7	87.1 ± 6.8	ns
EF [%]	119.1 ± 10.2	118.0 ± 9.5	ns
FS length [%]	174.9 ± 42.2	157.6 ± 21.6	ns
M-Mode			
LVAWd [%]	105.4 ± 11.3	110.7 ± 10.2	ns
LVPWd [%]	125.0 ± 7.9	113.8 ± 3.8	ns
LVIDd [%]	89.4 ± 5.7	90.1 ± 1.4	ns
LV Mass[%]	101.4 ± 12.0	98.4 ± 6.8	ns
PW-Doppler			
MV E [%]	66.6 ± 6.1	75.1 ± 2.4	ns
MV A [%]	68.5 ± 3.4	79.1 ± 4.2	ns
MV E/A [%]	97.1 ± 6.2	96.2 ± 4.0	ns
IVRT [%]	106.4 ± 8.2	96.6 ± 7.3	ns
IVCT [%]	142.2 ± 23.4	88.3 ± 8.5	*
AET [%]	93.0 ± 4.5	87.4 ± 3.6	ns
Tei-Index [%]	130.9 ± 15.9	115.6 ± 19.7	ns
Tissue-Doppler			
MV E’ [%]	88.2 ± 17.7	107.6 ± 17.6	ns
MV A’ [%]	73.9 ± 5.9	73.0 ± 6.8	ns
MV E’/A’ [%]	104.8 ± 19.1	164.2 ± 38.9	ns
MV E/E’ [%]	84.1 ± 9.9	82.6 ± 11.5	ns
S’ [%]	112.6 ± 5.1	101.4 ± 6.9	ns

Six- to seven-week-old *Isg15*^-/-^ mice were infected intraperitoneally with 10^5^ PFU/mouse of CVB3–1 or CVB3-39. Transthoracic echocardiography was performed one day prior to infection (baseline) and at day 3 (acute phase) immediately before sacrifice. Group sizes were as follows: baseline: CVB3–39 N = 6, CVB3–1 N = 8, d3: CVB3–39 N = 6 and CVB3–1 N = 8. Data were normalized as percentage change from baseline and normalized data were used for statistical comparisons between virus strains at day 3 using unpaired two-tailed t-tests or Mann–Whitney U tests when normality was not met. Statistical significance was defined as p ≤ 0.05 and is indicated by a single asterisk (*).

**Table 2:** Echocardiographic assessment in CVB3-1–infected mice.

CVB3-1	Baseline	Day 3	Baseline vs. day 3
LV Performance			
Heart rate [bpm]	418.2 ± 12.0	453.6 ± 18.4	ns
LVEDV [µl]	53.0 ± 1.5	36.3 ± 1.2	*
LVESV [µl]	27.2 ± 1.9	15.5 ± 1.6	ns
Stroke volume [µl]	25.9 ± 1.2	20.8 ± 1.9	*
Cardiac output [ml/min]	10.8 ± 0.5	9.4 ± 0.8	ns
EF [%]	49.1 ± 2.5	57.2 ± 4.4	ns
FS length [%]	6.3 ± 0.9	9.1 ± 1.2	ns
M-Mode			
LVAWd [mm]	0.8 ± 0.0	0.9 ± 0.1	ns
LVPWd [mm]	0.7 ± 0.0	0.8 ± 0.0	*
LVIDd [mm]	3.8 ± 0.1	3.4 ± 0.1	*
LV Mass [mg]	97.6 ± 4.7	94.4 ± 3.7	ns
PW-Doppler			
MV E [mm/s]	855.3 ± 28.1	640.0 ± 21.8	*
MV A [mm/s]	544.2 ± 18.2	426.7 ± 17.1	*
MV E/A	1.6 ± 0.0	1.5 ± 0.0	ns
IVRT [ms]	13.8 ± 0.6	13.1 ± 0.7	ns
IVCT [ms]	12.3 ± 0.7	10.7 ± 0.9	ns
AET [ms]	49.8 ± 2.1	43.2 ± 1.8	*
Tei-Index	0.5 ± 0.1	0.5 ± 0.0	ns
Tissue-Doppler			
MV E’ [mm/s]	-17.0 ± 2.7	-15.7 ± 1.5	ns
MV A’ [mm/s]	-29.7 ± 2.6	-20.6 ± 0.9	*
MV E’/A’	0.6 ± 0.1	0.8 ± 0.1	ns
MV E/E’	-63.0 ± 12.8	-43.3 ± 4.5	ns
S’ [mm/s]	27.0 ± 0.8	27.3 ± 1.9	ns

Six- to seven-week-old *Isg15*^-/-^ mice were infected intraperitoneally with 10^5^ PFU/mouse of CVB3-1. Transthoracic echocardiography was performed one day prior to infection (baseline) and at day 3 (acute phase) immediately before sacrifice. Group sizes were as follows: baseline: CVB3–1 N = 8, d3: and CVB3–1 N = 8. Data are shown as mean ± SEM. Changes in echocardiographic parameters within the same animal over time were analyzed using paired t-tests. Statistical significance was defined as p ≤ 0.05 and is indicated by a single asterisk (*).

**Table 3:** Echocardiographic assessment in CVB3-39–infected mice.

CVB3-39	Baseline	Day 3	Baseline vs. day 3
LV Performance			
Heart rate [bpm]	434.1 ± 14.7	435.8 ± 17.4	ns
LVEDV [µl]	51.8 ± 1.8	37.8 ± 5.2	*
LVESV [µl]	27.3 ± 2.2	17.0 ± 2.9	*
Stroke volume [µl]	24.6 ± 1.4	20.8 ± 2.7	ns
Cardiac output [ml/min]	10.7 ± 0.6	9.1 ± 1.4	ns
EF [%]	47.6 ± 3.0	55.4 ± 2.8	ns
FS length [%]	6.3 ± 0.8	9.6 ± 1.3	ns
M-Mode			
LVAWd [mm]	0.8 ± 0.0	0.9 ± 0.1	ns
LVPWd [mm]	0.7 ± 0.0	0.8 ± 0.0	*
LVIDd [mm]	3.7 ± 0.0	3.3 ± 0.2	ns
LV Mass [mg]	97.3 ± 2.1	98.6 ± 11.8	ns
PW-Doppler			
MV E [mm/s]	859.7 ± 27.2	567.4 ± 41.5	*
MV A [mm/s]	603.4 ± 34.1	410.6 ± 23.8	*
MV E/A	1.4 ± 0.1	1.4 ± 0.1	ns
IVRT [ms]	13.3 ± 0.6	13.9 ± 0.8	ns
IVCT [ms]	9.6 ± 1.1	12.5 ± 0.6	ns
AET [ms]	47.9 ± 1.4	44.5 ± 2.1	ns
Tei-Index	0.5 ± 0.0	0.6 ± 0.0	ns
Tissue-Doppler			
MV E’ [mm/s]	-19.0 ± 1.4	-15.7 ± 1.9	ns
MV A’ [mm/s]	-27.4 ± 1.4	-20.0 ± 1.5	*
MV E’/A’	0.8 ± 0.1	0.8 ± 0.1	ns
MV E/E’	-46.9 ± 4.3	-38.8 ± 4.9	ns
S’ [mm/s]	22.9 ± 1.5	25.6 ± 1.2	ns

Six- to seven-week-old *Isg15*^-/-^ mice were infected intraperitoneally with 10^5^ PFU/mouse of CVB3-39. Transthoracic echocardiography was performed one day prior to infection (baseline) and at day 3 (acute phase) immediately before sacrifice. Group sizes were as follows: baseline: CVB3–39 N = 6, d3: CVB3–39 N = 6. Data are shown as mean ± SEM. Changes in echocardiographic parameters within the same animal over time were analyzed using paired t-tests. Statistical significance was defined as p ≤ 0.05 and is indicated by a single asterisk (*).

Datasets comprising two independent factors (time and virus strain) were analyzed using two-way ANOVA (**Figures 1B–D, F–H**, **2A–G**, **3B–H**, **4A–C**, **5C, E–H, L**; **Supplementary Figures 1A–D**; **2A–C**). Where repeated measurements within the same animals were available, a mixed effect model (REML) was applied (**Figure 5B**). *Post hoc* comparisons were performed using Sidak’s multiple-comparison test. Comparisons of post-infection time points to baseline were conducted using one-way ANOVA followed by Dunnett’s multiple-comparison test (**Figures 1D, F–G**, **3B, F–H**, **4A–C**, **5C, E–H, L**; **Supplementary Figures 1A–D**, **2A–C**). Survival curves were analyzed using the log-rank (Mantel–Cox) test (**Figure 5A**), and ordinal or non-normally distributed data were evaluated using the Mann–Whitney U test (**Figures 1E**, **3A**, **5D, J**). Statistical significance was defined as p ≤ 0.05.

## Results

### Cardiomyocyte detargeting uncouples myocardial viral replication from systemic infection in *Isg15*-deficient mice.

To determine whether selective suppression of cardiac infection remains effective in the absence of ISG15, *Isg15^-/-^* mice were infected intraperitoneally with either the replication-competent control virus CVB3–39 or the cardiomyocyte-detargeted variant CVB3-1 (39). CVB3–39 contains target sequences for the *C. elegans*–specific microRNA miR-39, which is not expressed in mammalian tissues and therefore does not affect viral replication. In contrast, CVB3–1 carries target sequences for the muscle-enriched microRNA miR-1, resulting in selective suppression of viral replication in miR-1–expressing tissues, including cardiomyocytes and skeletal muscle cells, while preserving replication in non-muscle tissues (25). Myocardial viral burden was quantified during the acute phase of infection (day 3), when viral replication peaks, and at day 7 post infection (**Figure 1A**).

To determine whether selective suppression of cardiac infection remains effective in the absence of ISG15, *Isg15*^-/-^ mice were infected intraperitoneally with CVB3 containing miR-39 target sites present only in *C. elegans* (CVB3-39) or the cardiomyocyte-detargeted variant CVB3–1 containing miR-1 target sites (25, 39). Myocardial viral burden was quantified during the acute phase of infection (day 3) and later on at day 7 post infection, when inflammatory injury peaks in the infected mouse heart (**Figure 1A**). In *Isg15*^-/-^ mice infected with the control virus CVB3-39, high levels of infectious virus were detected in cardiac tissue at day 3 and remained elevated at day 7, indicating sustained myocardial replication (**Figure 1B**). In contrast, infection with the miR-1–detargeted virus CVB3–1 resulted in a marked reduction of infectious viral titers already during the acute phase. This difference became even more pronounced by day 7, when viral titers in CVB3-1–infected hearts were drastically reduced, reaching the limit of detection (**Figure 1B**). Quantification of viral genome copy numbers by RT-qPCR closely paralleled infectious titers and likewise demonstrated significantly reduced viral genome abundance in CVB3-1–infected mice at both time points (**Figure 1C**). Together, these findings demonstrate that miR-1–mediated detargeting remains fully operative in *Isg15*^-/-^ mice and enables experimental uncoupling of local cardiac viral replication from systemic infection.

We next examined whether suppression of myocardial replication translated into protection from cardiac injury. In *Isg15*^-/-^ mice infected with CVB3-39, circulating cardiac troponin levels were markedly elevated, indicating substantial cardiomyocyte injury already at day 3 post infection (**Figure 1D**). Histological analysis revealed inflammatory infiltrates and myocyte damage consistent with viral myocarditis, which became particularly prominent by day 7 (**Figure 1E**). In contrast, CVB3-1–infected *Isg15*^-/-^ mice displayed troponin levels comparable to uninfected controls and preserved myocardial architecture without detectable inflammatory lesions (**Figures 1D, E**). To quantify inflammatory cell recruitment into cardiac tissue, cardiac immune cell populations were analyzed by flow cytometry (**Figures 1F, G**). Total leukocyte numbers remained low and comparable between infection groups at day 3 (**Figure 1G**). By day 7, however, CVB3-39–infected *Isg15*^-/-^ exhibited pronounced accumulation of cardiac immune cells, whereas leukocyte numbers in CVB3-1–infected animals remained near baseline levels (**Figure 1G**). The infiltrating immune cell compartment was dominated by myeloid cells. In particular, Ly6C^high^ inflammatory monocytes represented the most abundant population, consistent with the characteristic inflammatory response during viral myocarditis (40) (**Figure 1F**). Smaller populations of neutrophils and dendritic cells were additionally detected (**Figure 1F**). Quantitative analysis confirmed markedly reduced infiltration of myeloid immune cells in CVB3-1–infected mice compared with CVB3–39 infection at day 7 (**Figure 1F**). Other lymphoid populations, including B cells, NK cells, and T cells, remained comparatively sparse and did not exhibit consistent differences between infection groups (**Supplementary Figure 1**).

To further characterize cardiac inflammatory responses at the transcriptional level, myocardial expression of antiviral and inflammatory genes was analyzed. At day 3 post infection, robust induction of interferon-responsive genes (*Ifn-b* and the interferon-stimulated gene *Ifit3*), proinflammatory cytokines (*Il-1b* and *Tnf-a*), and chemokines (*Ccl2* and *Cxcl10*) was observed in both infection groups (**Figure 1H**). Notably, the magnitude of this early transcriptional response remained largely comparable between CVB3–39 and CVB3–1 infection despite marked differences in myocardial viral burden. By day 7, overall expression levels declined, but clear differences between virus groups emerged. Hearts from CVB3-39–infected mice maintained elevated expression of antiviral and inflammatory mediators, whereas expression levels in CVB3-1–infected animals were markedly reduced, consistent with the absence of sustained myocardial viral replication (**Figure 1B**) and inflammatory cell infiltration (**Figure 1G**). Taken together, these findings demonstrate that myocardial injury and myocarditis in *Isg15*^-/-^ mice remain strictly dependent on local viral replication. Cardiomyocyte detargeting effectively prevents myocardial inflammation and tissue injury, thereby enabling experimental separation of local cardiac pathology from systemic disease manifestations.

### Peripheral viral dissemination remains preserved following cardiomyocyte detargeting.

Having established that cardiomyocyte detargeting effectively suppresses myocardial viral replication in *Isg15^-/-^* mice, we next examined whether cardiac attenuation alters systemic viral dissemination during acute infection (day 3) and at day 7 post infection. The exocrine pancreas represents the primary replication hub of CVB3 following intraperitoneal infection (30). Accordingly, both virus strains produced high pancreatic viral titers at day 3 post infection that declined toward day 7 (**Figure 2A**). Importantly, pancreatic viral titers remained comparable between CVB3-39– and CVB3-1–infected mice at both time points. We next assessed viral burden in the liver, a major secondary target organ contributing to systemic antiviral and metabolic responses during CVB3 infection (4, 10, 41). Infectious hepatic viral titers were similar between CVB3-39– and CVB3-1–infected mice at day 3 and declined toward day 7, with a trend toward lower titers in CVB3-1–infected animals during the later phase of infection (**Figure 2B**). Quantification of viral genome copy numbers by RT-qPCR closely mirrored infectious titers and did not reveal biologically relevant differences between virus strains at either time point (**Figure 2C**). Skeletal muscle was analyzed as an additional miR-1–expressing tissue to assess potential off-target effects of the detargeting strategy (42). Viral replication was quantified in musculus quadriceps femoris and gastrocnemius. In both muscles, infectious viral titers remained overall low and declined from day 3 to day 7 post infection (**Figures 2D, F**). Infectious titers and viral genome copy numbers were largely comparable between CVB3–39 and CVB3–1 infection during the acute phase (**Figures 2D–G**). By day 7, modestly lower viral titers were observed in CVB3-1–infected mice, particularly in the gastrocnemius muscle, consistent with previously described minor off-target effects of the detargeting strategy (25) (**Figures 2F, G**). Taken together, suppression of myocardial viral replication did not substantially alter systemic viral dissemination in *Isg15^-/-^* mice, indicating that cardiomyocyte detargeting preserves overall systemic infection dynamics.

### Acute hepatitis develops independently of myocardial viral replication.

Beyond the pancreas, the liver represents a major target organ during systemic CVB3 infection and substantially contributes to antiviral immunity, systemic inflammation, and metabolic reprogramming (10). We therefore examined whether suppression of myocardial viral replication influences hepatic injury in *Isg15^-/-^* mice. Histopathological analysis revealed a severe acute hepatitis during early infection. At day 3 post infection, hematoxylin–eosin staining demonstrated multifocal inflammatory infiltrates and hepatocellular injury in both CVB3-39– and CVB3-1–infected mice (**Figure 3A**). Histological scoring confirmed comparable severity of hepatic inflammation between infection groups (**Figure 3A**). Although inflammatory lesions declined toward day 7, histological scores remained elevated, indicating persistence of hepatic injury into the subacute phase. Consistent with histological liver injury, serum concentrations of aspartate aminotransferase (AST), alanine aminotransferase (ALT), and lactate dehydrogenase (LDH) were markedly elevated during the acute phase and declined toward near-baseline levels by day 7 (**Figure 3B**). In parallel, circulating albumin concentrations were reduced during infection, consistent with impaired hepatic synthetic function (**Figure 3B**). Biochemical manifestations of hepatic injury were unaffected despite effective suppression of myocardial viral replication.

To further characterize hepatic antiviral and inflammatory responses, expression of interferon-stimulated genes, cytokines, and chemokines was quantified in liver tissue. At day 3 post infection, *Ifn-b*, *Ifit3*, the proinflammatory cytokines *Il-1b* and *Tnf-a*, as well as the chemokines *Ccl2* and *Cxcl10* were strongly induced (**Figures 3C–E**). Expression levels declined markedly toward day 7, reflecting resolution of the acute inflammatory response. Thus, hepatic antiviral and inflammatory signaling developed independently of myocardial viral replication. Consistent with the observed chemokine induction, acute infection was characterized by pronounced hepatic infiltration of myeloid immune cells at day 3, dominated by Ly6C^high^ inflammatory monocytes and monocyte-derived macrophages, whereas resident Kupffer cells were transiently reduced (**Figures 3F–H**). By day 7, infiltrating myeloid populations declined toward baseline levels. NK cells remained elevated throughout infection, whereas B-cell populations became more prominent at day 7, with slightly increased numbers observed in CVB3-1–infected mice (**Figure 3F**). Dendritic cells and T cells were likewise expanded during infection, whereas neutrophil accumulation remained comparatively modest (**Supplementary Figure 2**). Taken together, these findings demonstrate that severe acute hepatitis develops independently of myocardial viral replication in *Isg15^-/-^* CVB3 infection. Despite markedly enhanced cardiac viral replication and myocarditis in CVB3-39–infected mice, suppression of myocardial infection did not attenuate hepatic inflammation or liver injury.

### Systemic inflammatory activation and early cardiac dysfunction occur independently of myocardial viral replication.

To further characterize systemic inflammatory responses during infection, circulating interferons, cytokines, and chemokines were quantified in serum. During the acute phase of infection (day 3), serum concentrations IFN-a, IFN-b, and TNF-a were markedly elevated and accompanied by increased levels of IL-6, IL-10, CCL2, CCL5, CXCL1, and CXCL10, indicating robust systemic immune activation (**Figures 4A–C**). Cytokine concentrations declined toward baseline levels by day 7. Although IL-6 and IL-10 levels were moderately increased in CVB3-39–infected mice during the acute phase, overall systemic cytokine profiles remained largely comparable between CVB3–39 and CVB3–1 infection, indicating that enhanced myocardial viral replication and myocarditis do not substantially amplify systemic inflammatory signaling.

Systemic inflammatory activation has previously been linked to early cardiac functional alterations during CVB3 infection (13, 25, 43). We therefore assessed cardiac performance by echocardiography during the acute phase of infection. Both virus variants induced reductions in end-diastolic volume and stroke volume, with comparable alterations observed in CVB3-39– and CVB3-1–infected mice (**Figures 4D, E**). Global cardiac performance parameters, including cardiac output and ejection fraction, likewise did not differ substantially between infection groups at this stage of infection (**Figures 4F, G**). Thus, early cardiac functional alterations developed largely independently of myocardial viral replication, consistent with the overall comparable magnitude of systemic inflammatory responses between CVB3-39– and CVB3-1–infected mice.

### Systemic metabolic deterioration develops independently of myocardial viral replication.

*Isg15* deficiency during CVB3 infection has previously been linked to profound metabolic disturbances (10, 12, 16, 19, 20). We therefore next investigated whether suppression of myocardial viral replication influences systemic metabolic deterioration during infection. Both CVB3-39– and CVB3-1–infected *Isg15*^-/-^ mice developed severe systemic disease associated with progressive body weight loss and reduced survival (**Figures 5A, B**). Body weight declined rapidly following infection and continued to decrease toward day 7 post infection without substantial differences between virus groups, consistent with a pronounced systemic catabolic state. To further characterize systemic metabolic alterations, circulating glucose concentrations and hepatic glycogen stores were assessed. Both infection groups developed marked hypoglycemia during acute infection that persisted at low levels throughout the observation period (**Figure 5C**). Periodic acid–Schiff staining demonstrated profound depletion of hepatic glycogen stores during the acute phase of infection (**Figure 5D**). Partial recovery of glycogen content was observed at later time points in subsets of animals, although substantial inter-individual variability remained. Together, these findings indicate severe systemic disruption of glucose homeostasis independent of myocardial viral replication.

Consistent with the systemic catabolic phenotype, marked depletion of adipose tissue was observed during infection. Inguinal white adipose tissue (iWAT) mass was substantially reduced already at day 3 and declined further toward day 7 in both infection groups (**Figure 5E**). Likewise, skeletal muscle wasting developed progressively during infection. Weights of musculus quadriceps femoris and gastrocnemius were markedly reduced, particularly at day 7 post infection, whereas cardiac mass exhibited only comparatively modest alterations (**Figures 5F–H**). These findings identify skeletal muscle and adipose tissue as principal targets of systemic wasting during *Isg15*-deficient CVB3 infection. To determine whether skeletal muscle wasting was associated with inflammatory myositis, histological analyses were performed at baseline and during infection (days 3 and 7). Hematoxylin–eosin staining revealed mild inflammatory changes in skeletal muscle, reflected by modest increases in myositis score and muscle inflammation score (MIS) at day 3 that remained slightly elevated at day 7, albeit with substantial inter-individual variability (**Figures 5I, J**). F4/80 staining demonstrated a modest increase in macrophage signal during the acute phase selectively in CVB3-39–infected mice (**Figures 5K, L**). Despite this localized increase in macrophage infiltration, overall inflammatory involvement of skeletal muscle remained mild (**Figures 5I, J**). Taken together, these findings demonstrate that systemic metabolic deterioration during *Isg15*-deficient CVB3 infection develops independently of myocardial viral replication. Suppression of myocarditis did not attenuate hypoglycemia, glycogen depletion, adipose tissue loss, skeletal muscle wasting, or overall disease severity.

## Discussion

Our study demonstrates that aggravated myocarditis and systemic wasting can be experimentally dissociated during *Isg15*-deficient CVB3 infection. Cardiomyocyte-directed suppression of viral replication effectively prevented myocardial injury, inflammatory cell infiltration, and histological myocarditis, yet did not substantially attenuate inflammatory activation, metabolic deterioration, early cardiac dysfunction, or mortality. These findings indicate that the pronounced wasting phenotype observed in *Isg15*-deficient mice is not primarily driven by aggravated myocardial infection but instead reflects broader defects in antiviral and immunometabolic adaptation during severe enteroviral disease. Importantly, the present study does not exclude that systemic antiviral and inflammatory dysregulation caused by global *Isg15* deficiency contributes to aggravation of myocardial pathology. Because *Isg15* deficiency affects antiviral responses across multiple organs and cell types, including interferon responses, viral clearance, and metabolic homeostasis (10–12), both local and peripheral mechanisms likely cooperate in shaping myocarditis severity in this setting.

A central unresolved question in *Isg15*-deficient CVB3 infection has been whether aggravated myocarditis secondarily amplifies organismal disease progression. In *Isg15*-deficient mice, elevated cardiac viral titers coincide with inflammatory activation, metabolic collapse, and impaired cardiac performance (10, 11), raising the possibility that aggravated myocardial infection amplifies systemic disease. This possibility appeared particularly plausible in light of previous findings showing impaired mitochondrial ATP production capacity, altered glucose handling, and cardiac atrophy in *Isg15*-deficient hearts during CVB3 infection (Bredow et al, 2024). Such energetic decompensation of infected myocardium could theoretically increase systemic metabolic demand and thereby contribute to infection-associated wasting. By selectively suppressing cardiomyocyte infection while preserving overall infection dynamics (25), our study directly addressed this hypothesis experimentally. Despite near-complete elimination of myocardial viral replication and myocarditis in CVB3-1–infected mice, peripheral organ infection, inflammatory activation, metabolic deterioration, and mortality remained largely unaffected. Together, these findings argue against a model in which metabolically decompensating myocarditis acts as a major driver of infection-associated wasting in *Isg15*-deficient mice. The functional data further support this interpretation. Reductions in ventricular filling and cardiac output occurred independently of myocardial viral burden and histological myocarditis, indicating that early echocardiographic alterations primarily reflect inflammatory and circulatory adaptation to severe infection rather than myocarditis-dependent pump failure. This interpretation is consistent with previous work demonstrating sustained systolic contractility despite reduced cardiac output during acute CVB3 infection, supporting a model in which early hemodynamic impairment reflects altered vascular tone, reduced preload, and hypovolemia rather than intrinsic contractile dysfunction (25).

The liver emerged as a central organ within this disease axis. Beyond serving as a major target organ of CVB3 infection (41), the liver integrates antiviral signaling with metabolic homeostasis (4, 10). Previous studies demonstrated that hepatocyte type I interferon signaling limits viral cytotoxicity and dissemination (10), while ISGylation accelerates viral clearance from liver and spleen (4). In our study, suppression of myocardial viral replication did not attenuate hepatic viral burden, acute hepatitis, hepatic inflammatory gene expression, or liver-infiltrating myeloid cell accumulation. Unchanged hepatic inflammation despite prevention of myocarditis therefore indicates that extra-cardiac antiviral failure is sufficient to sustain disease progression during *Isg15*-deficient infection. Previous work further identified ISG15 as a central regulator of infection-induced metabolic response. During CVB3 infection, mice develop hypoglycemia with reduced hepatic glycogen stores, and *Isg15*-deficient animals show impaired glucose homeostasis already at baseline and during infection (10). Mechanistically, ISG15 promotes oxidative metabolic adaptation and supports ATP-demanding gluconeogenesis during inflammatory stress (10). Our data extend this concept by showing that hypoglycemia and hepatic glycogen depletion persist even when myocarditis is prevented. Thus, the metabolic phenotype in *Isg15*-deficient mice cannot be explained by aggravated myocardial injury but instead reflects organism-wide failure of infection-induced metabolic adaptation.

The wasting phenotype should therefore be viewed as part of this organism-wide immunometabolic failure. *Isg15*-deficient mice developed progressive weight loss, adipose tissue depletion, skeletal muscle wasting, persistent hypoglycemia, and reduced survival irrespective of myocardial involvement. Previous work using Seahorse-based analysis demonstrated that ISG15 reduces glucose demand, supports mitochondrial ATP production capacity during infection, and counteracts cardiac atrophy and dysfunction (12). ISGylation of glycolytic enzymes further restrains glycolytic flux, thereby limiting inefficient glucose utilization during nutrient shortage (12, 18, 19). Because this metabolic reprogramming has already been comprehensively defined, the present study instead isolated a different variable, selective myocardial viral replication, within a shared *Isg15*-deficient background. In the absence of ISG15 dependent adaption, increased energetic demand likely enhances reliance on endogenous substrate mobilization, including muscle protein breakdown. Pronounced skeletal muscle loss despite only mild local myositis supports a systemic catabolic mechanism rather than primary inflammatory destruction of muscle tissue. Although modest reductions in skeletal muscle viral titers were observed at day 7 following CVB3–1 infection, consistent with minor miR-1–mediated off-target attenuation, these differences were not accompanied by preservation of skeletal muscle mass or relevant differences in inflammatory muscle pathology. This further supports the concept that systemic inflammatory and metabolic responses, rather than local viral muscle infection or aggravated myocarditis, represent the dominant drivers of infection-associated wasting in *Isg15*-deficient mice. Inflammatory mediators including TNF-a and IL-6 likely further promote adipose tissue lipolysis, proteolytic pathway activation, and substrate mobilization during severe infection (44–46). Together, these findings support a model in which defective ISG15-dependent antiviral adaptation drives organism-wide metabolic stress characterized by persistent substrate mobilization and progressive tissue wasting during CVB3 infection rather than the phenotype arising from the myocardial infection itself.

The hypoglycemia observed in the present study should be interpreted in the context of acute systemic CVB3 infection, which is characterized by severe viral pancreatitis, systemic inflammation, and a pronounced catabolic state. Following intraperitoneal infection, CVB3 rapidly infects pancreatic acinar cells, impairing digestive enzyme production and nutrient absorption, while infected animals also exhibit reduced food intake as part of the acute disease response (29, 47). Together, these changes limit nutrient availability and increase metabolic demand, resulting in transient hypoglycemia. In contrast, persistent CVB3 infection has been associated with pancreatic β-cell infection, impaired insulin secretion, and hyperglycemia at later stages of disease (48–51) indicating that acute hypoglycemia and chronic hyperglycemia represent distinct stages of CVB3-induced metabolic dysfunction.

Several limitations should be considered. First, the present study focused on the acute phase of infection and therefore does not address whether aggravated myocarditis contributes to later cardiac remodeling or chronic functional deterioration. Second, systemic *Isg15-*deficiency affects multiple organs and immune cell populations simultaneously, precluding precise dissection of organ-specific contributions to disease progression. Finally, miR-1–mediated detargeting induced mild attenuation of viral replication in peripheral muscle tissue apart from cardiac tissue. However, these effects remained quantitatively small and were not associated with relevant alterations in inflammatory or metabolic disease manifestations.

In conclusion, our findings establish that aggravated myocarditis and systemic wasting can be experimentally uncoupled during *Isg15*-deficient CVB3 infection. While myocardial viral replication is essential for local myocarditis, inflammatory, metabolic, and functional deterioration primarily reflect organism-wide defects in antiviral and immunometabolic adaptation rather than myocarditis-driven amplification of disease.

## Data Availability

The raw data supporting the conclusions of this article will be made available by the authors, without undue reservation.

## References

[1] Schneider WM Chevillotte MD Rice CM . Interferon-stimulated genes: a complex web of host defenses. Annu Rev Immunol. (2014) 32:513–45. doi: 10.1146/annurev-immunol-032713-120231 24555472 PMC4313732

[2] Althof N Harkins S Kemball CC Flynn CT Alirezaei M Whitton JL . *In vivo* ablation of type I interferon receptor from cardiomyocytes delays coxsackieviral clearance and accelerates myocardial disease. J Virol. (2014) 88:5087–99. doi: 10.1128/JVI.00184-14 24574394 PMC3993796

[3] Deonarain R Cerullo D Fuse K Liu PP Fish EN . Protective role for interferon-beta in coxsackievirus B3 infection. Circulation. (2004) 110:3540–3. doi: 10.1161/01.CIR.0000136824.73458.20 15249500

[4] Koestner W Spanier J Klause T Tegtmeyer PK Becker J Herder V . Interferon-beta expression and type I interferon receptor signaling of hepatocytes prevent hepatic necrosis and virus dissemination in coxsackievirus B3-infected mice. PloS Pathog. (2018) 14:e1007235. doi: 10.1371/journal.ppat.1007235 30075026 PMC6107283

[5] Wessely R Klingel K Knowlton KU Kandolf R . Cardioselective infection with coxsackievirus B3 requires intact type I interferon signaling: implications for mortality and early viral replication. Circulation. (2001) 103:756–61. doi: 10.1161/01.cir.103.5.756 11156890

[6] Wang J Lu W Zhang J Du Y Fang M Zhang A . Loss of Trim29 mitigates viral myocarditis by attenuating Perk-driven ER stress response in male mice. Nat Commun. (2024) 15:3481. doi: 10.1038/s41467-024-44745-x 38664417 PMC11045800

[7] Fang M Zhang A Du Y Lu W Wang J Minze LJ . Trim18 is a critical regulator of viral myocarditis and organ inflammation. J BioMed Sci. (2022) 29:55. doi: 10.1186/s12929-022-00840-z 35909127 PMC9339186

[8] Althof N Goetzke CC Kespohl M Voss K Heuser A Pinkert S . The immunoproteasome‐specific inhibitor ONX 0914 reverses susceptibility to acute viral myocarditis. EMBO Mol Med. (2018) 10:200. doi: 10.15252/EMMM.201708089 29295868 PMC5801517

[9] Perng YC Lenschow DJ . Isg15 in antiviral immunity and beyond. Nat Rev Microbiol. (2018) 16:423–39. doi: 10.1038/S41579-018-0020-5 29769653 PMC7097117

[10] Kespohl M Bredow C Klingel K Voss M Paeschke A Zickler M . Protein modification with ISG15 blocks coxsackievirus pathology by antiviral and metabolic reprogramming. Sci Adv. (2020) 6:eaay1109. doi: 10.1126/sciadv.aay1109 32195343 PMC7065878

[11] Rahnefeld A Klingel K Schuermann A Diny NL Althof N Lindner A . Ubiquitin-like protein ISG15 (interferon-stimulated gene of 15 kDa) in host defense against heart failure in a mouse model of virus-induced cardiomyopathy. Circulation. (2014) 130:1589–600. doi: 10.1161/circulationaha.114.009847 25165091

[12] Bredow C Thery F Wirth EK Ochs S Kespohl M Kleinau G . ISG15 blocks cardiac glycolysis and ensures sufficient mitochondrial energy production during coxsackievirus B3 infection. Cardiovasc Res. (2024) 120:644–57. doi: 10.1093/CVR/CVAE026 38309955 PMC11074791

[13] Kelm N Kespohl M Smagurauskaite G Vales S Karuppanan K Mburu P . Assessing customized multivalent chemokine-binding peptide treatment in a murine model of coxsackievirus B3 myocarditis. Basic Res Cardiol. (2025) 120:393. doi: 10.1007/S00395-025-01098-W 40009121 PMC11976344

[14] Ketscher L Hannß R Moralese DJ Basters A Guerra S Goldmann T . Selective inactivation of USP18 isopeptidase activity *in vivo* enhances ISG15 conjugation and viral resistance. PNAS. (2015) 112:1577–82. doi: 10.1073/pnas.1412881112 PMC4321242 25605921

[15] Thery F Eggermont D Impens F . Proteomics mapping of the ISGylation landscape in innate immunity. Front Immunol. (2021) 12:720765. doi: 10.3389/fimmu.2021.720765 34447387 PMC8383068

[16] Baldanta S Fernandez-Escobar M Acin-Perez R Albert M Camafeita E Jorge I . ISG15 governs mitochondrial function in macrophages following vaccinia virus infection. PloS Pathog. (2017) 13:e1006651. doi: 10.1371/journal.ppat.1006651 29077752 PMC5659798

[17] Zhang Y Thery F Wu NC Luhmann EK Dussurget O Foecke M . The *in vivo* ISGylome links ISG15 to metabolic pathways and autophagy upon Listeria monocytogenes infection. Nat Commun. (2019) 10:5383. doi: 10.1038/S41467-019-13393-X 31772204 PMC6879477

[18] Yan S Kumari M Xiao H Jacobs C Kochumon S Jedrychowski M . IRF3 reduces adipose thermogenesis via ISG15-mediated reprogramming of glycolysis. J Clin Invest. (2021) 131. doi: 10.1172/JCI144888 33571167 PMC8011904

[19] Eggermont D Eggermont L Aslantaş N Thery F Boucher K Beling A . Proteomics and tracer metabolomics link GAPDH ISGylation to glycolytic control. Genome Biol. (2026) 27(1):135. doi: 10.1186/s13059-026-04034-w 41814427 PMC13093933

[20] Alcalá S Sancho P Martinelli P Navarro D Pedrero C Martín-Hijano L . ISG15 and ISGylation is required for pancreatic cancer stem cell mitophagy and metabolic plasticity. Nat Commun. (2020) 11:2682. doi: 10.1038/s41467-020-16395-2 32472071 PMC7260233

[21] Liu H Wang S Wang J Guo X Song Y Fu K . Energy metabolism in health and diseases. Signal Transduct Target Ther. (2025) 10:69. doi: 10.1038/s41392-025-02141-x 39966374 PMC11836267

[22] Sagar S Liu PP Cooper LT Jr. Myocarditis. Lancet. (2012) 379:738–47. doi: 10.1016/s0140-6736(11)60648-x 22185868 PMC5814111

[23] Fairweather D Rose NR . Inflammatory heart disease: a role for cytokines. Lupus. (2005) 14:646–51. doi: 10.1191/0961203305LU2192OA 16218459

[24] Esfandiarei M McManus BM . Molecular biology and pathogenesis of viral myocarditis. Annu Rev Pathol. (2008) 3:127–55. doi: 10.1146/ANNUREV.PATHMECHDIS.3.121806.151534 18039131

[25] Ochs S Pinkert S Borowski S Huis in ’t Veld LGM Kelm N Geisler A . Dissecting myocardial and systemic drivers of cardiac dysfunction in the murine CVB3 myocarditis model using microRNA-guided viral detargeting. Basic Res Cardiol. (2026) 121(4):775–99. doi: 10.1007/s00395-026-01187-4 42213155 PMC13372866

[26] He F Yao H Wang J Xiao Z Xin L Liu Z . Coxsackievirus B3 engineered to contain microRNA targets for muscle-specific microRNAs displays attenuated cardiotropic virulence in mice. J Virol. (2014) 89:908. doi: 10.1128/JVI.02933-14 25339771 PMC4300646

[27] Xiao Z He F Feng M Liu Z Liu Z Li S . Engineered coxsackievirus B3 containing multiple organ-specific miRNA targets showed attenuated viral tropism and protective immunity. Infect Genet Evol. (2022) 103:105316. doi: 10.1016/j.meegid.2022.105316 35718333

[28] Osiak A Utermöhlen O Niendorf S Horak I Knobeloch K-P . ISG15, an interferon-stimulated ubiquitin-like protein, is not essential for STAT1 signaling and responses against vesicular stomatitis and lymphocytic choriomeningitis virus. Mol Cell Biol. (2005) 25:6338. doi: 10.1128/MCB.25.15.6338-6345.2005 16024773 PMC1190360

[29] Pinkert S Kespohl M Kelm N Kaya Z Heuser A Klingel K . Exploration of analgesia with tramadol in the coxsackievirus B3 myocarditis mouse model. Viruses. (2021) 13:1222. doi: 10.3390/V13071222 34202636 PMC8310306

[30] Pinkert S Pryshliak M Pappritz K Knoch K Hazini A Dieringer B . Development of a new mouse model for coxsackievirus-induced myocarditis by attenuating coxsackievirus B3 virulence in the pancreas. Cardiovasc Res. (2020) 116:1756–66. doi: 10.1093/CVR/CVZ259 31598635

[31] Bauer M Cheng S Jain M Ngoy S Theodoropoulos C Trujillo A . Echocardiographic speckle-tracking based strain imaging for rapid cardiovascular phenotyping in mice. Circ Res. (2011) 108:908. doi: 10.1161/CIRCRESAHA.110.239574 21372284 PMC3376717

[32] Zacchigna S Paldino A Falc I Daskalopoulos EP Dal Ferro M Vodret S . Towards standardization of echocardiography for the evaluation of left ventricular function in adult rodents: a position paper of the ESC Working Group on Myocardial Function. Cardiovasc Res. (2020) 117(1):43–59. doi: 10.1093/cvr/cvaa110 32365197

[33] Gallage S Avila JEB Ramadori P Focaccia E Rahbari M Ali A . A researcher’s guide to preclinical mouse NASH models. Nat Metab. (2022) 4:1632–49. doi: 10.1038/s42255-022-00700-y 36539621

[34] Szalay G Sauter M Hald J Weinzierl A Kandolf R Klingel K . Sustained nitric oxide synthesis contributes to immunopathology in ongoing myocarditis attributable to interleukin-10 disorders. Am J Pathol. (2006) 169:2085–93. doi: 10.2353/ajpath.2006.060350 17148671 PMC1762471

[35] Veteläinen RL Bennink RJ de Bruin K van Vliet A van Gulik TM . Hepatobiliary function assessed by 99mTc-mebrofenin cholescintigraphy in the evaluation of severity of steatosis in a rat model. Eur J Nucl Med Mol Imaging. (2006) 33:1107–14. doi: 10.1007/s00259-006-0125-3 16738848

[36] Santos FM Dias RS de Oliveira MD Costa ICTA Fernandes LDS Pessoa CR . Animal model of arthritis and myositis induced by the Mayaro virus. PloS Negl Trop Dis. (2019) 13:e0007375. doi: 10.1371/JOURNAL.PNTD.0007375 31050676 PMC6519846

[37] Afzali AM Ruck T Wiendl H Meuth SG . Animal models in idiopathic inflammatory myopathies: how to overcome a translational roadblock? Autoimmun Rev. (2017) 16:478–94. doi: 10.1016/J.AUTREV.2017.03.001 28286105

[38] Stach LM Huis in ‘t Veld LGM Schaub T Jendrach M Ornaghi M Schwabenland M . Decoding IFN-mediated immunity and cell dynamics in viral encephalitis: insights from coxsackievirus B3 infection. iScience. (2026) 29:114324. doi: 10.1016/J.ISCI.2025.114324 41503211 PMC12768882

[39] Elsner L Hinze L Hazini A Heimann L Geisler A Dieringer B . Improved safety of new microRNA-regulated oncolytic coxsackievirus B3 observed after intravenous administration in colorectal-tumor-bearing mice. Viruses. (2026) 18:143. doi: 10.3390/v18010143 41600904 PMC12846641

[40] Musigk N Suwalski P Golpour A Fairweather D Klingel K Martin P . The inflammatory spectrum of cardiomyopathies. Front Cardiovasc Med. (2024) 11:1251780. doi: 10.3389/fcvm.2024.1251780 38464847 PMC10921946

[41] Kimura T Flynn CT Whitton JL . Hepatocytes trap and silence coxsackieviruses, protecting against systemic disease in mice. Commun Biol. (2020) 3:580. doi: 10.1038/s42003-020-01303-7 33067530 PMC7568585

[42] Isakova A Fehlmann T Keller A Quake SR . A mouse tissue atlas of small noncoding RNA. Proc Natl Acad Sci USA. (2020) 117:25634–45. doi: 10.1073/pnas.2002277117 32978296 PMC7568261

[43] Kespohl M Goetzke CC Althof N Bredow C Kelm N Pinkert S . TF-FVIIa PAR2-β-arrestin signaling sustains organ dysfunction in coxsackievirus B3 infection of mice. Arterioscler Thromb Vasc Biol. (2024) 44:843–65. doi: 10.1161/ATVBAHA.123.320157 38385286

[44] Cawthorn WP Sethi JK . TNF-alpha and adipocyte biology. FEBS Lett. (2008) 582:117–31. doi: 10.1016/j.febslet.2007.11.051 18037376 PMC4304634

[45] Makki K Froguel P Wolowczuk I . Adipose tissue in obesity-related inflammation and insulin resistance: cells, cytokines, and chemokines. ISRN Inflammation. (2013) 2013:139239. doi: 10.1155/2013/139239 24455420 PMC3881510

[46] Webster JM Kempen L Hardy RS Langen RCJ . Inflammation and skeletal muscle wasting during cachexia. Front Physiol. (2020) 11:597675. doi: 10.3389/fphys.2020.597675 33329046 PMC7710765

[47] Mena I Fischer C Gebhard JR Perry CM Harkins S Whitton JL . Coxsackievirus infection of the pancreas: evaluation of receptor expression, pathogenesis, and immunopathology. Virology. (2000) 271:276–88. doi: 10.1006/VIRO.2000.0332 10860882

[48] Chehadeh W Kerr-Conte J Pattou F Alm G Lefebvre J Wattre P . Persistent infection of human pancreatic islets by coxsackievirus B is associated with alpha interferon synthesis in beta cells. J Virol. (2000) 74:10153–64. doi: 10.1128/jvi.74.21.10153-10164.2000 11024144 PMC102054

[49] Dotta F Censini S van Halteren AG Marselli L Masini M Dionisi S . Coxsackie B4 virus infection of beta cells and natural killer cell insulitis in recent-onset type 1 diabetic patients. Proc Natl Acad Sci USA. (2007) 104:5115–20. doi: 10.1073/pnas.0700442104 17360338 PMC1829272

[50] Gallagher GR Brehm MA Finberg RW Barton BA Shultz LD Greiner DL . Viral infection of engrafted human islets leads to diabetes. Diabetes. (2015) 64:1358–69. doi: 10.2337/db14-1020 25392246 PMC4375078

[51] Carre A Vecchio F Flodstrom-Tullberg M You S Mallone R . Coxsackievirus and type 1 diabetes: Diabetogenic mechanisms and implications for prevention. Endocr Rev. (2023) 44:737–51. doi: 10.1210/endrev/bnad007 36884282

