# Supplementary material for: Systemic inflammatory and metabolic disease progression persists despite prevention of myocarditis in Isg15-deficient Coxsackievirus B3 infection
Source: Front Immunol. 2026 Aug 11;17:1907453. doi: 10.3389/fimmu.2026.1907453 (PMC13503590; doi:10.3389/fimmu.2026.1907453)
Supplement: Supplementary file 1 [file DataSheet1.pdf]

SUPPLEMENTARY FILE

Myocardial viral replication is dispensable for systemic inflammatory and metabolic disease progression in Coxsackievirus B3 infection in *Isg15*-deficient mice

Sarah Ochs[1], Sandra Pinkert[1,2], Sophia Borowski[1,2], Lisa GM Huis in 't Veld[1], Nicolas Kelm[1,3], Ziya Kaya[4,5], Anne Hausen[6], Karin Klingel[7], Matthias M. Gaida[6,7,8], Antje Beling[1,2*]

1 – Charité – Universitätsmedizin Berlin, corporate member of Freie Universität Berlin and Humboldt-Universität zu Berlin, Institute of Biochemistry, 10117 Berlin, Germany
2 – Deutsches Zentrum für Herz-Kreislauf-Forschung, partner site Berlin, 10117 Berlin, Germany
3 – Department of Medicine I, Department of Gastroenterology and Hepatology, Faculty of Medicine and University Hospital Carl Gustav Carus, TU Dresden University of Technology, 01069 Dresden, Germany.
4 – Kardiologie, Angiologie und Pneumologie, Medizinische Klinik für Innere Medizin III, Universitätsklinikum Heidelberg, 69120 Heidelberg, Germany.
5 – Deutsches Zentrum für Herz-Kreislauf-Forschung (DZHK), Partner Site Heidelberg, 69120 Heidelberg, Germany.
6 – Institute of Pathology, University Medical Center Mainz, Johannes-Gutenberg-Universität Mainz, 55131 Mainz, Germany
7 – Cardiopathology, Institute for Pathology and Neuropathology, University Hospital Tübingen, 72076 Tübingen, Germany
8– TRON, Translational Oncology at the University Medical Center of the Johannes Gutenberg University Mainz, 55131 Mainz, Germany
9 – Research Center for Immunotherapy, University Medical Center Mainz, Johannes-Gutenberg-Universität Mainz, 55131 Mainz, Germany

[*]**Corresponding author**
Prof. Antje Beling
Phone: 0049 30 450 528 187
Email: antje.beling@charite.de
Charité – Universitätsmedizin Berlin
Institute of Biochemistry
Charitéplatz 1
10117 Berlin
Germany

ORCID ID: 0000-0002-1826-5248

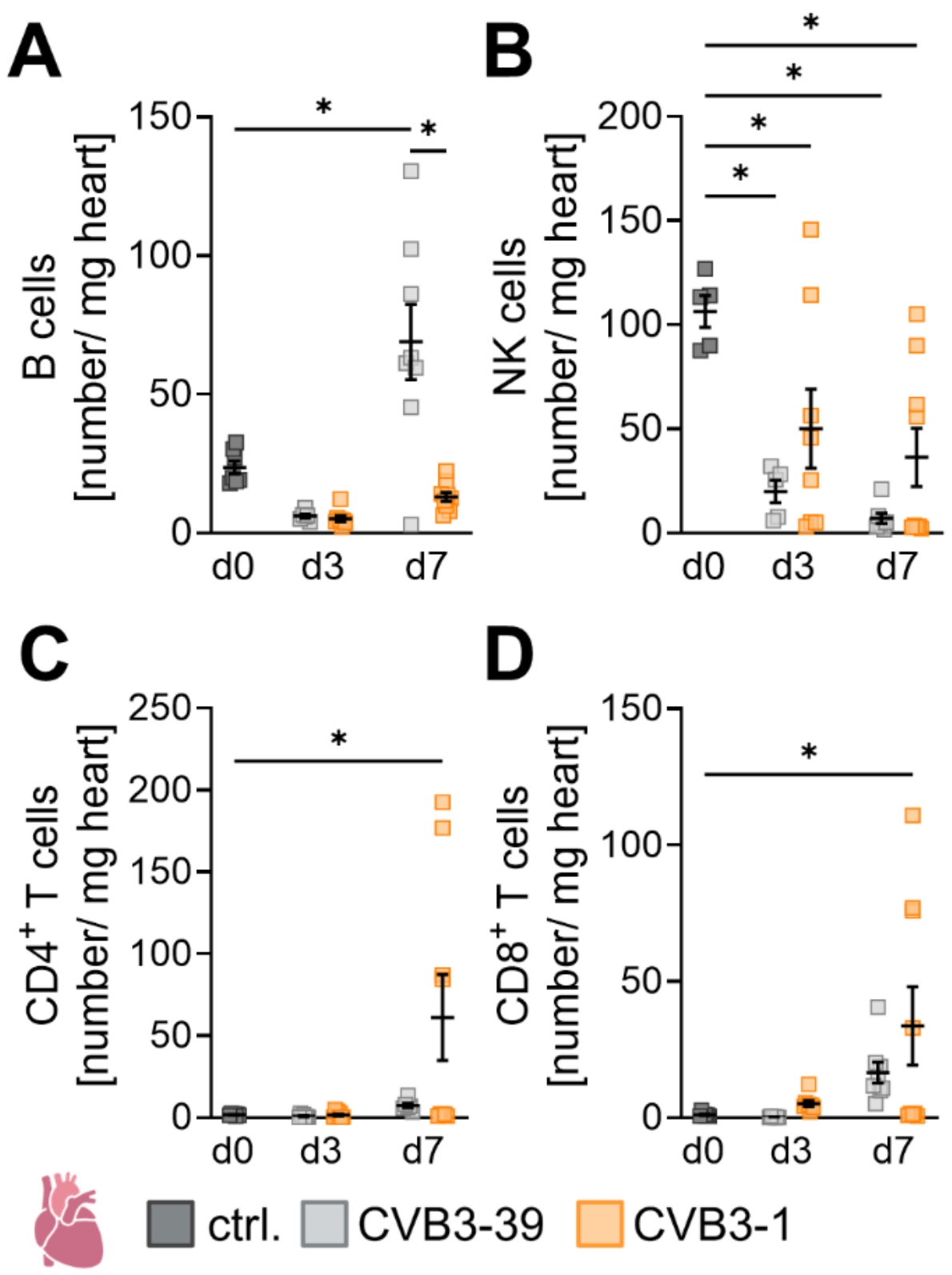

Figure S1. Cardiac lymphoid immune cell infiltration during CVB3 infection. Six- to seven-week-old ISG15$^{-/-}$ mice were infected intraperitoneally with 10^5 PFU/mouse of CVB3-39 or CVB3-1 and heart was analyzed at day 3 (acute phase) and day 7 (subacute phase). Age-matched uninfected mice served as baseline controls (day 0). Group sizes: d0 (N = 8), d3 CVB3-39 (N = 9), d3 CVB3-1 (N = 9), d7 CVB3-39 (N = 10), d7 CVB3-1 (N = 10). (A-C) Immune cell populations were analyzed by flow cytometry and cell numbers per milligram of heart tissue are shown for the following populations: $CD45^+CD11b^-NK1.1^-B220^+CD3^-$ B cells (A), $CD45^+CD11b^-B220^+CD3^-NK1.1^+$ NK cells (B), $CD45^+CD11b^-B220^-CD3^+CD4^+CD8^-$ T helper cells (C) and $CD45^+CD11b^-B220^-CD3^+CD4^-CD8^+$ cytotoxic T lymphocytes (D) Data are shown as mean ± SEM. Outliers were detected using the ROUT (Q=1%) method. Two-way ANOVA with Sidak's post hoc test was used for datasets with two independent factors (time and virus strain). One-way ANOVA with Dunnett's post hoc test compared post-infection time points to baseline. Statistical significance was defined as $p \leq 0.05$ (*).

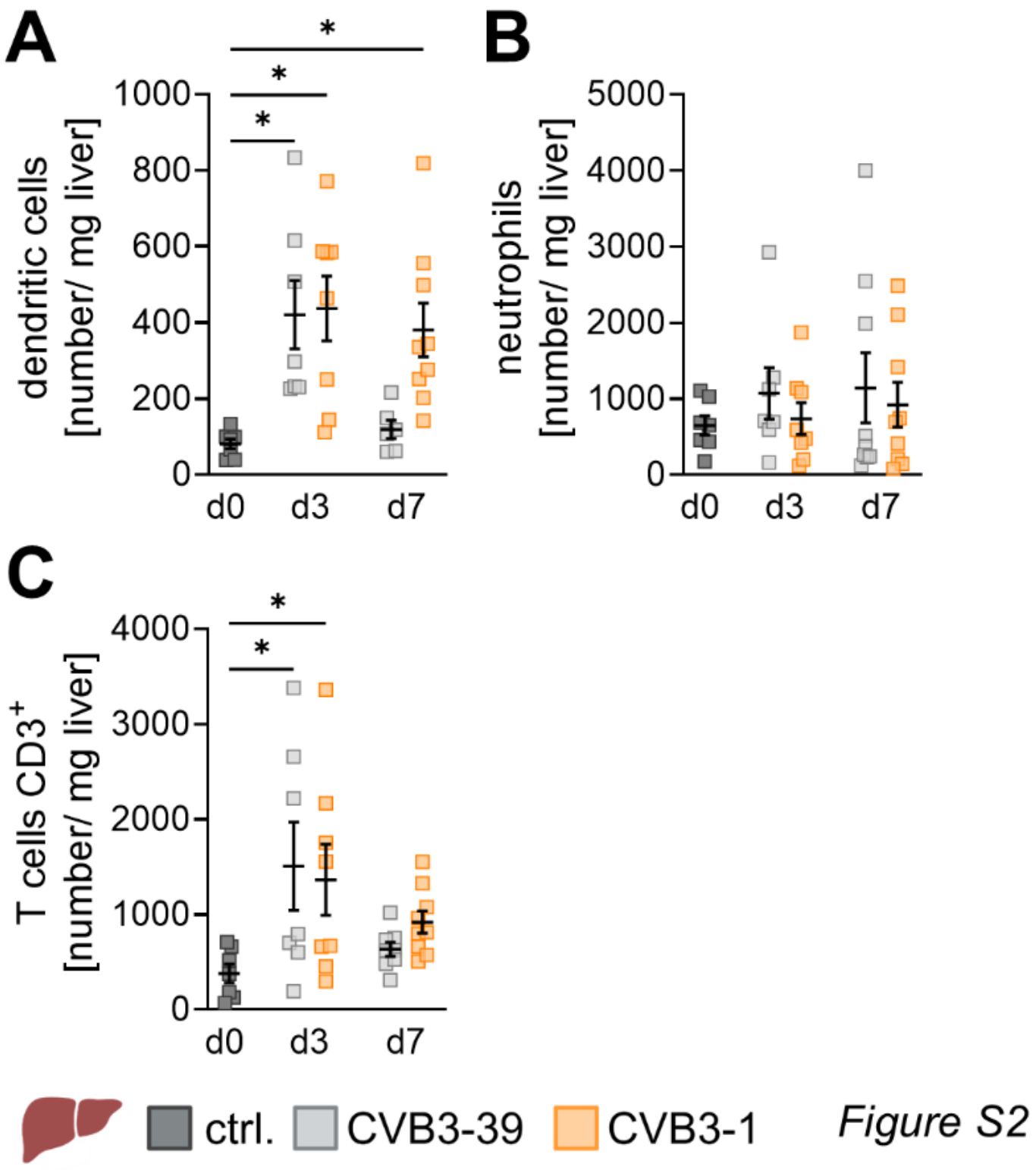

Figure S2. Hepatic lymphoid and myeloid immune cell infiltration during CVB3 infection. Six- to seven-week-old ISG15$^{-/-}$ mice were infected intraperitoneally with 10^5 PFU/mouse of CVB3-39 or CVB3-1 and liver was analyzed at day 3 (acute phase) and day 7 (subacute phase). Age-matched uninfected mice served as baseline controls (day 0). Group sizes: d0 (N = 8), d3 CVB3-39 (N = 9), d3 CVB3-1 (N = 9), d7 CVB3-39 (N = 10), d7 CVB3-1 (N = 10). (A-C) Immune cell populations were analyzed by flow cytometry and cell numbers per milligram of liver tissue are shown for the following populations: CD45$^+$lineage$^-$CD11b$^+$Ly6G$^-$F4/80$^-$CD11c$^+$MHC-II$^+$ dendritic cells (A), CD45$^+$lineage$^-$CD11b$^+$Ly6G$^+$ neutrophils (B) and CD45$^+$CD19$^-$CD3$^+$ T cells (C). Data are shown as mean ± SEM. Outliers were detected using the ROUT (Q=1%) method. Two-way ANOVA with Sidak's post hoc test was used for datasets with two independent factors (time and virus strain). One-way ANOVA with Dunnett's post hoc test compared post-infection time points to baseline. Statistical significance was defined as $p \leq 0.05$ (*).

Liver - myeloid

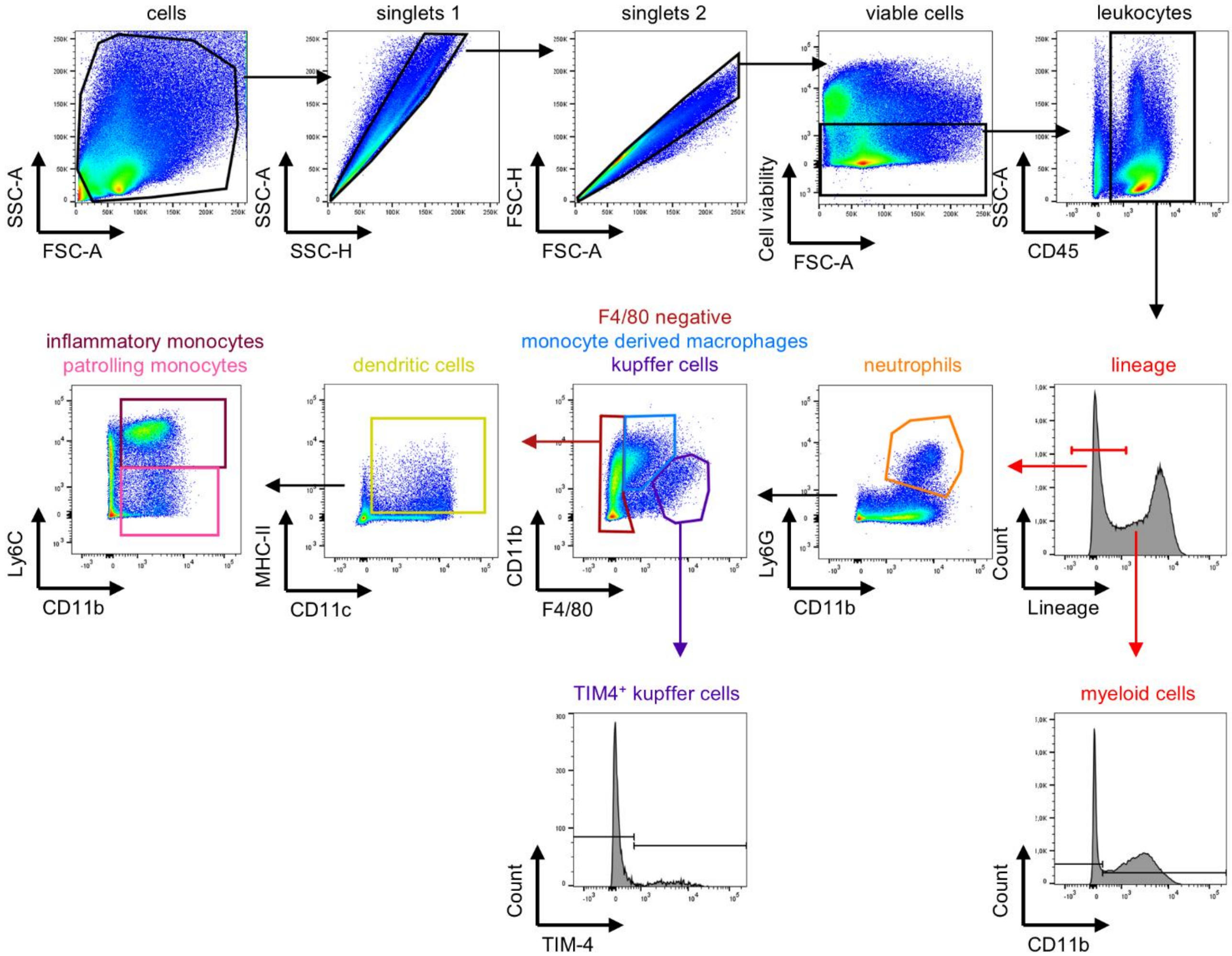

leukocytes:	CD45^{+}
lineage:	CD45^{+}CD3^{-}B220^{-}Ter119^{-}CD49b^{-}
myeloid cells:	CD45^{+}lineage^{-}CD11b^{+}
neutrophils:	CD45^{+}lineage^{-}CD11b^{+}Ly6G^{+}
monocyte derived macrophages (MoMF):	CD45^{+}lineage^{-}CD11b^{high}Ly6G^{-}F4/80^{intermediate}
kupffer cells:	CD45^{+}lineage^{-}CD11b^{intermediate}Ly6G^{-}F4/80^{high}
kupffer cells TIM4^{+}:	CD45^{+}lineage^{-}CD11b^{intermediate}Ly6G^{-}F4/80^{high}TIM4^{+}
dendritic cells (DC):	CD45^{+}lineage^{-}CD11b^{+}Ly6G^{-}F4/80^{-}CD11c^{+}MHC-II^{+}
inflammatory monocytes:	CD45^{+}lineage^{-}CD11b^{+}Ly6G^{-}F4/80^{-}CD11c^{-}MHC-II^{-}Ly6C^{high}
patrolling monocytes:	CD45^{+}lineage^{-}CD11b^{+}Ly6G^{-}F4/80^{-}CD11c^{-}MHC-II^{-}Ly6C^{low}

Figure S3. Representative gating strategy identifying hepatic myeloid immune cell populations during infection by flow cytometry. Liver-derived cells were stained with a multi-parameter panel of fluorochrome-conjugated antibodies against lymphoid and myeloid surface markers and analyzed by flow cytometry. The gating strategy began with the exclusion of debris and doublets using FSC-A/SSC-A, SSC-A/SSC-H, and FSC-A/FSC-H scatter plots, followed by the removal of non-viable cells based on viability dye uptake. Leukocytes were then selected and refined through sequential exclusion of lymphoid and non-myeloid cells using a lineage marker dump channel. Distinct myeloid cell subsets were subsequently resolved using defined marker combinations. Full details of the gating criteria applied to each population are provided beneath the representative plots.

Liver - myeloid – schematic gating strategy

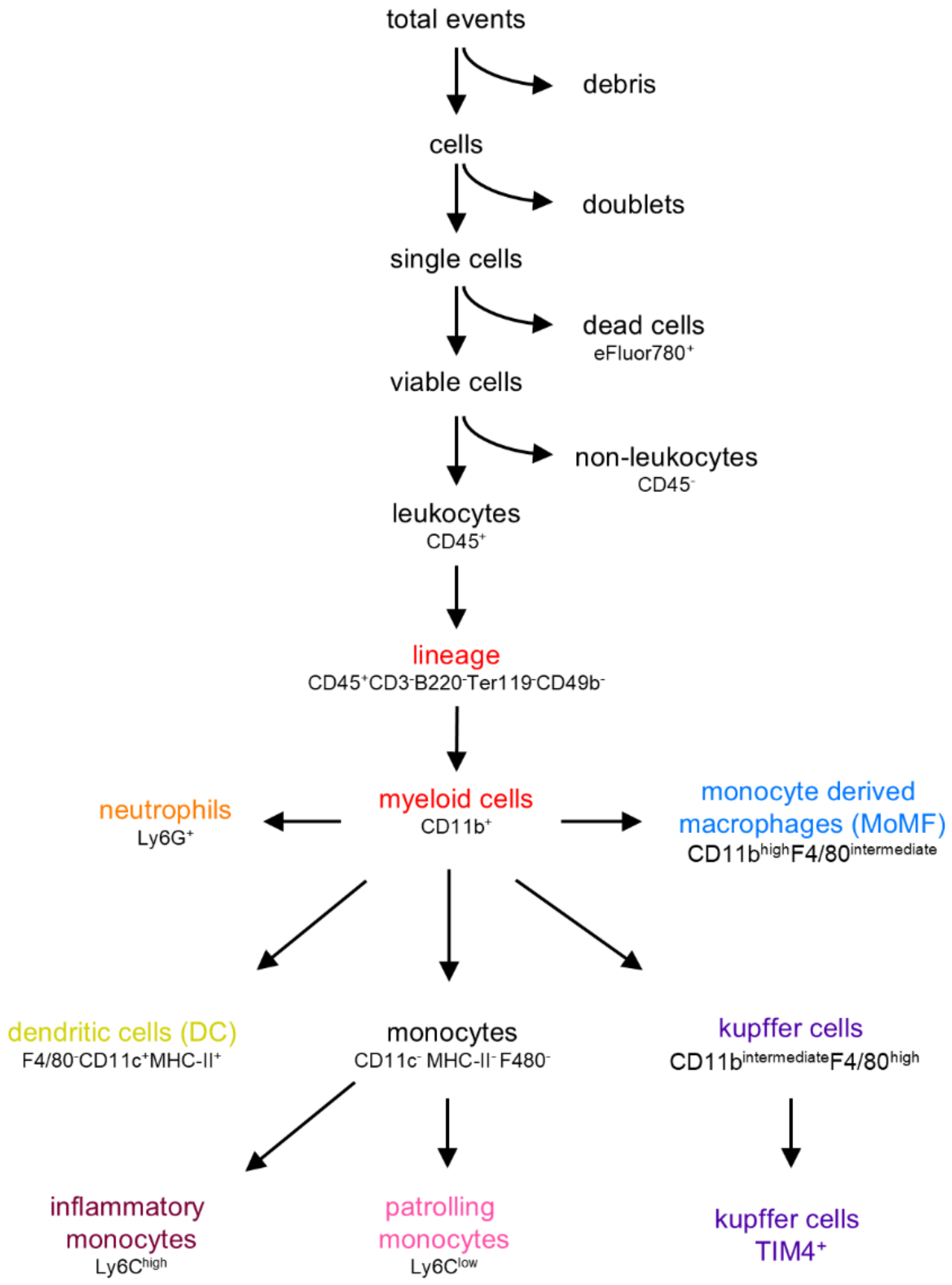

leukocytes:	CD45^{+}
lineage:	CD45^{+}CD3^{-}B220^{-}Ter119^{-}CD49b^{-}
myeloid cells:	CD45^{+}lineage^{-}CD11b^{+}
neutrophils:	CD45^{+}lineage^{-}CD11b^{+}Ly6G^{+}
monocyte derived macrophages (MoMF):	CD45^{+}lineage^{-}CD11b^{high}Ly6G^{-}F4/80^{intermediate}
kupffer cells:	CD45^{+}lineage^{-}CD11b^{intermediate}Ly6G^{-}F4/80^{high}
kupffer cells TIM4^{+}:	CD45^{+}lineage^{-}CD11b^{intermediate}Ly6G^{-}F4/80^{high}TIM4^{+}
dendritic cells (DC):	CD45^{+}lineage^{-}CD11b^{+}Ly6G^{-}F4/80^{-}CD11c^{+}MHC-II^{+}
inflammatory monocytes:	CD45^{+}lineage^{-}CD11b^{+}Ly6G^{-}F4/80^{-}CD11c^{-}MHC-II^{-}Ly6C^{high}
patrolling monocytes:	CD45^{+}lineage^{-}CD11b^{+}Ly6G^{-}F4/80^{-}CD11c^{-}MHC-II^{-}Ly6C^{low}

Figure S4. Schematic overview of the flow cytometry gating hierarchy for hepatic myeloid immune cell populations. This diagram depicts the sequential gating strategy employed in the flow cytometry analysis, providing a condensed summary of the representative gating plots shown in Figure S3. The specific marker combinations and gating criteria used to define each immune cell population are indicated beneath the schematic.

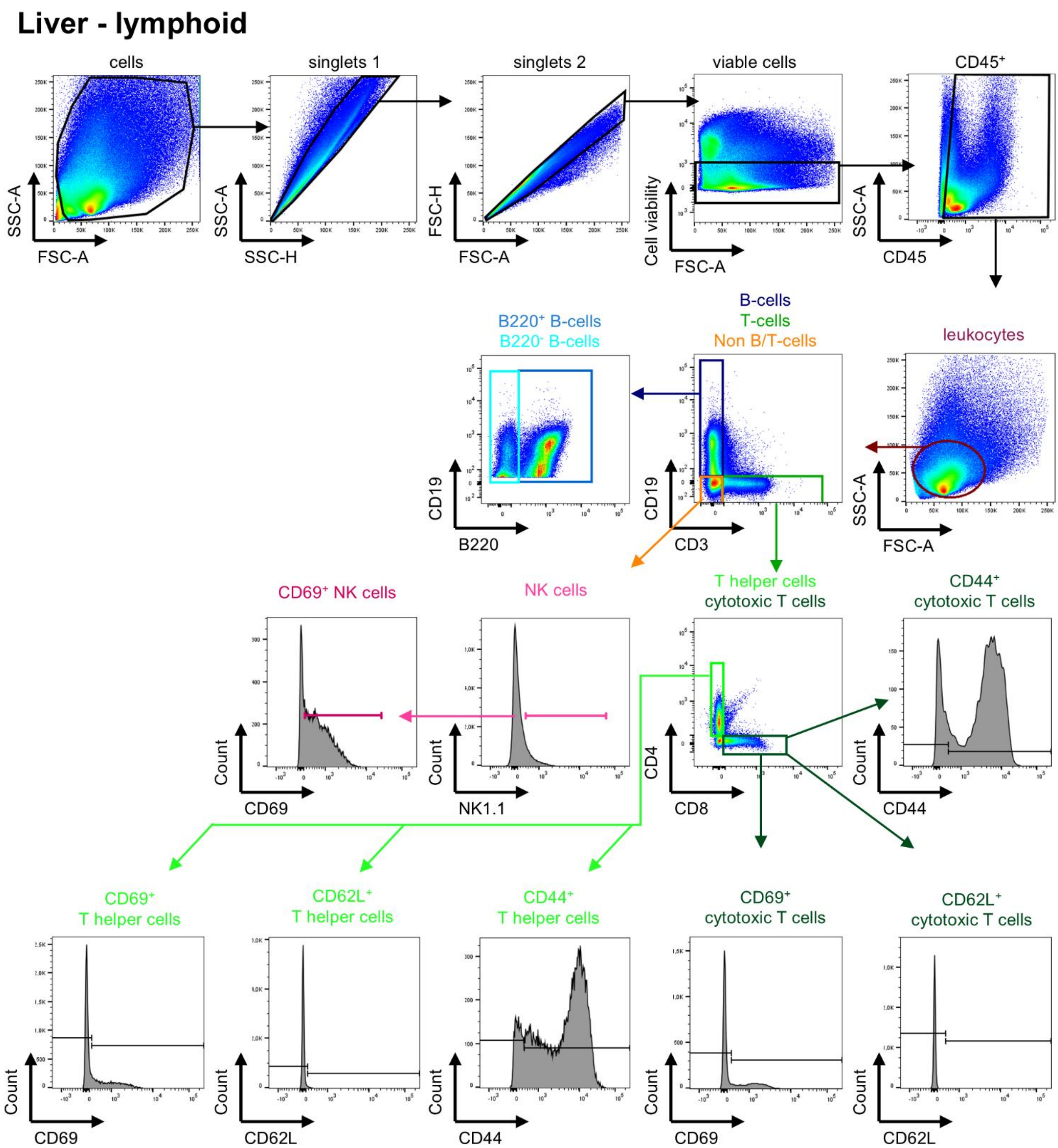

leukocytes:	$CD45^+$	T-cells:	$CD45^+CD19^-CD3^+$
B-cells:	$CD45^+CD19^+$	T helper cells:	$CD45^+CD19^-CD3^+CD8^-CD4^+$
B-cells B220+:	$CD45^+CD19^+B220^+$	T helper cells activation marker:	
B-cells B220-:	$CD45^+CD19^+B220^-$	CD69+:	amount of $CD45^+CD19^-CD3^+CD8^-CD4^+$ cells
		CD62L+:	amount of $CD45^+CD19^-CD3^+CD8^-CD4^+$ cells
Natural killer (NK) cells:	$CD45^+CD19^-CD3^-NK1.1^+$	CD44+:	amount of $CD45^+CD19^-CD3^+CD8^-CD4^+$ cells
NK cells CD69+:	$CD45^+CD19^-CD3^-NK1.1^+$	cytotoxic T cells:	$CD45^+CD19^-CD3^+CD4^-CD8^+$
		Cytotoxic T cells activation marker:	
		CD69+:	amount of $CD45^+CD19^-CD3^+CD4^-CD8^+$ cells
		CD62L+:	amount of $CD45^+CD19^-CD3^+CD4^-CD8^+$ cells
		CD44+:	amount of $CD45^+CD19^-CD3^+CD4^-CD8^+$ cells

Figure S5. Representative gating strategy for identifying hepatic lymphoid immune cell populations during infection by flow cytometry. Liver-derived cells were stained with a multi-parameter panel of fluorochrome-conjugated antibodies against lymphoid surface markers and

analyzed by flow cytometry. The gating strategy began with the exclusion of debris and doublets using FSC-A/SSC-A, SSC-A/SSC-H, and FSC-A/FSC-H scatter plots, followed by the removal of non-viable cells based on viability dye uptake. Leukocytes were subsequently isolated, and lymphoid cell subsets were resolved using defined marker combinations. Full details of the gating criteria applied to each population are provided beneath the representative plots.

Liver - lymphoid – schematic gating strategy

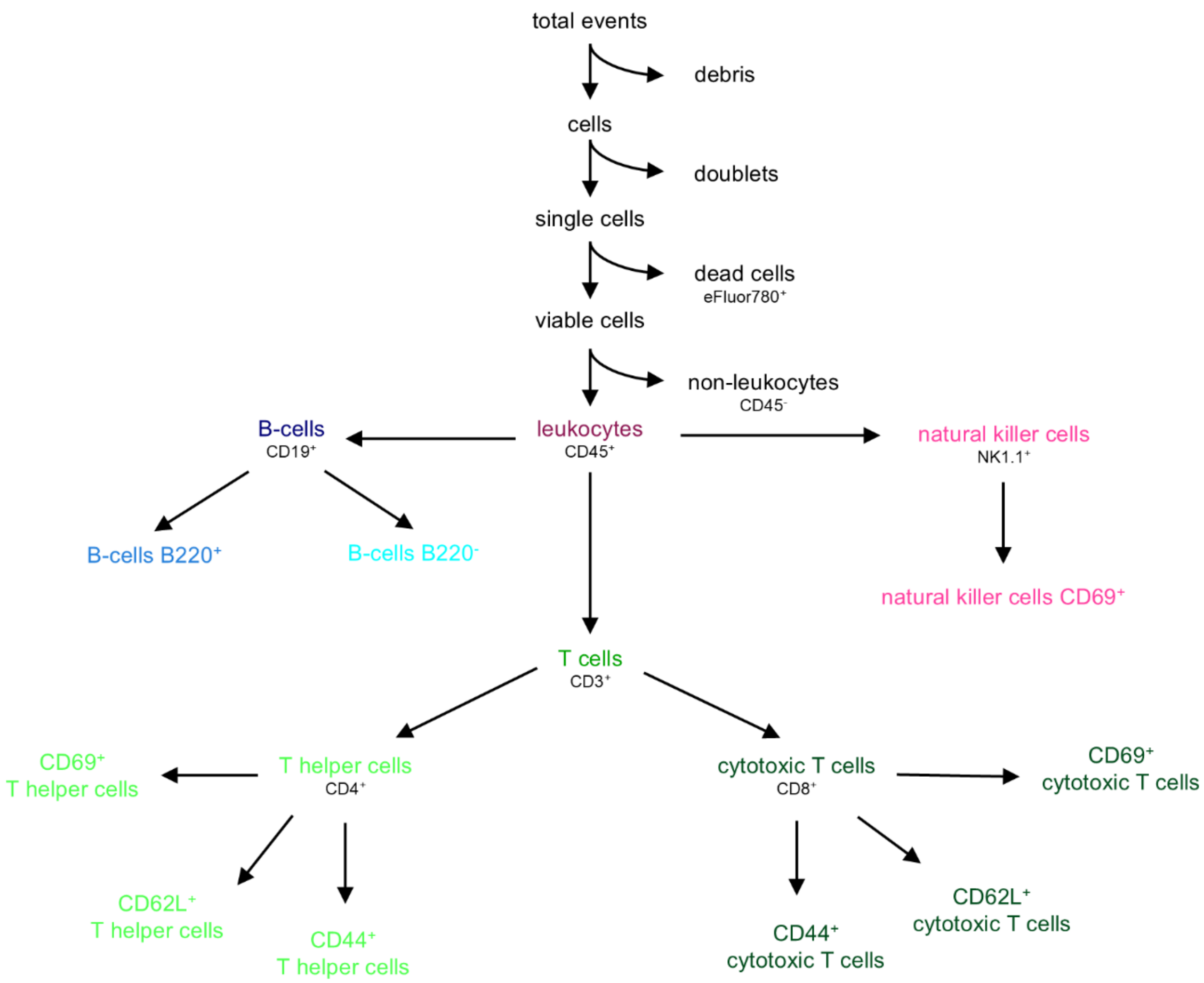

leukocytes:	$CD45^{+}$
B-cells:	$CD45^{+}CD19^{+}$
B-cells $B220^{+}$:	$CD45^{+}CD19^{+}B220^{+}$
B-cells $B220^{-}$:	$CD45^{+}CD19^{+}B220^{-}$
T-cells:	$CD45^{+}CD19^{-}CD3^{+}$
T helper cells:	$CD45^{+}CD19^{-}CD3^{+}CD8^{-}CD4^{+}$
T helper cells activation marker:	
$CD69^{+}$:	amount of $CD45^{+}CD19^{-}CD3^{+}CD8^{-}CD4^{+}$ cells
$CD62L^{+}$:	amount of $CD45^{+}CD19^{-}CD3^{+}CD8^{-}CD4^{+}$ cells
$CD44^{+}$:	amount of $CD45^{+}CD19^{-}CD3^{+}CD8^{-}CD4^{+}$ cells
cytotoxic T cells:	$CD45^{+}CD19^{-}CD3^{+}CD4^{-}CD8^{+}$
Cytotoxic T cells activation marker:	
$CD69^{+}$:	amount of $CD45^{+}CD19^{-}CD3^{+}CD4^{-}CD8^{+}$ cells
$CD62L^{+}$:	amount of $CD45^{+}CD19^{-}CD3^{+}CD4^{-}CD8^{+}$ cells
$CD44^{+}$:	amount of $CD45^{+}CD19^{-}CD3^{+}CD4^{-}CD8^{+}$ cells
Natural killer (NK) cells:	$CD45^{+}CD19^{-}CD3^{-}NK1.1^{+}$
NK cells $CD69^{+}$:	$CD45^{+}CD19^{-}CD3^{-}NK1.1^{+}$

Figure S6. Schematic overview of the flow cytometry gating hierarchy for hepatic lymphoid immune cell populations. This diagram depicts the sequential gating strategy employed in the flow cytometry analysis, providing a condensed summary of the representative gating plots shown in Figure S5. The specific marker combinations and gating criteria used to define each immune cell population are indicated beneath the schematic.

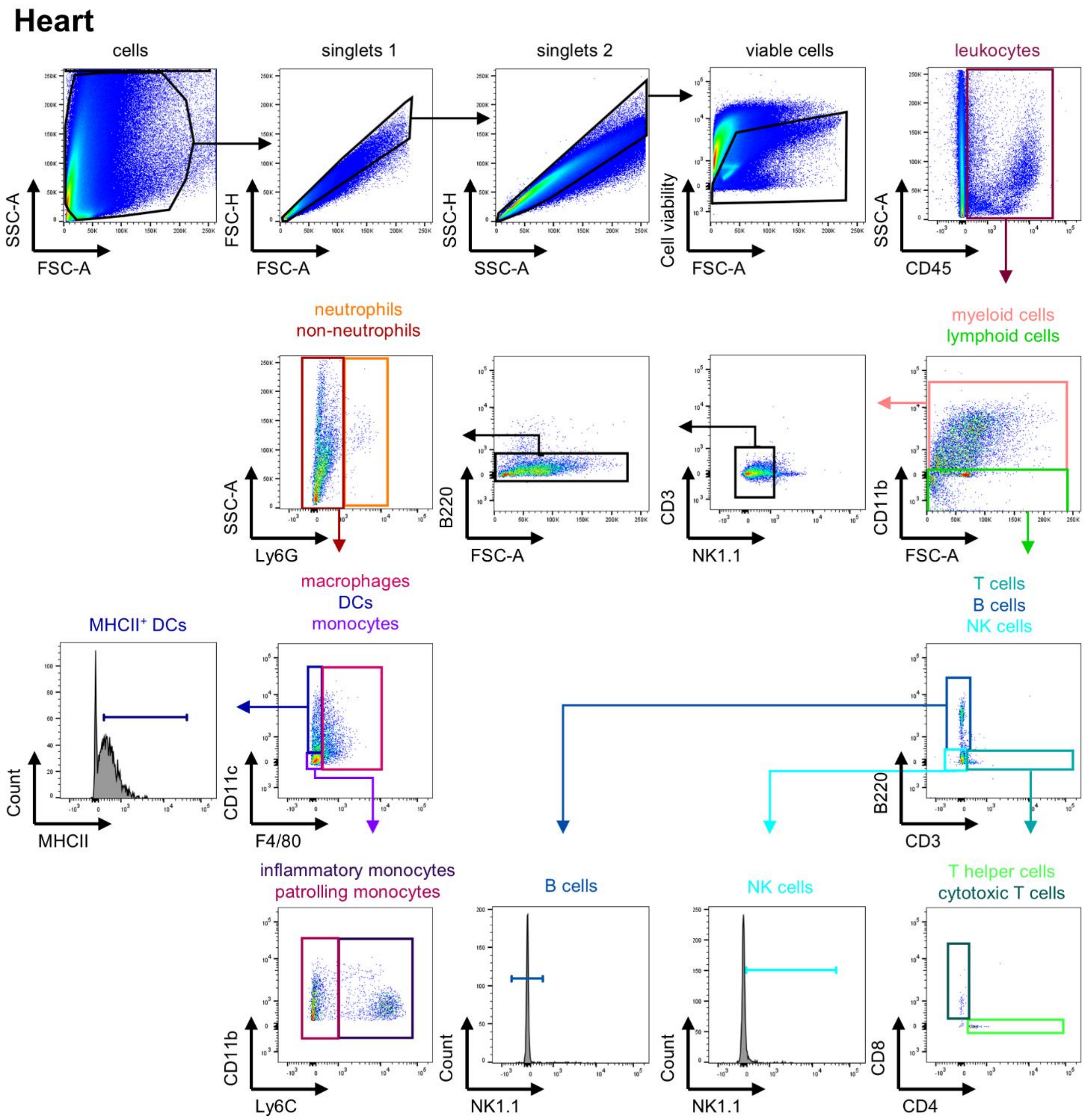

leukocytes:	CD45$^+$
myeloid cells:	CD45$^+$CD11b$^+$
neutrophils:	CD45$^+$CD11b$^+$CD3$^-$NK1.1$^-$B220$^-$Ly6G$^+$
macrophages:	CD45$^+$CD11b$^+$CD3$^-$NK1.1$^-$B220$^-$Ly6G$^-$CD11c$^{+/-}$F4/80$^+$
dendritic cells (DCs):	CD45$^+$CD11b$^+$CD3$^-$NK1.1$^-$B220$^-$Ly6G$^-$CD11c$^+$F4/80$^-$
MHCII$^+$ dendritic cells (DCs):	CD45$^+$CD11b$^+$CD3$^-$NK1.1$^-$B220$^-$Ly6G$^-$CD11c$^+$F4/80$^-$MHCII$^+$
monocytes:	CD45$^+$CD11b$^+$CD3$^-$NK1.1$^-$B220$^-$Ly6G$^-$CD11c$^-$F4/80$^-$
inflammatory monocytes:	CD45$^+$CD11b$^+$CD3$^-$NK1.1$^-$B220$^-$Ly6G$^-$CD11c$^-$F4/80$^-$Ly6C^{high}
patrolling monocytes:	CD45$^+$CD11b$^+$CD3$^-$NK1.1$^-$B220$^-$Ly6G$^-$CD11c$^-$F4/80$^-$Ly6C^{low}
lymphoid cells:	CD45$^+$CD11b$^-$
B cells:	CD45$^+$CD11b$^-$NK1.1$^-$B220$^+$CD3$^-$
T cells:	CD45$^+$CD11b$^-$B220$^-$CD3$^+$
T helper cells:	CD45$^+$CD11b$^-$B220$^-$CD3$^+$CD4$^+$CD8$^-$
cytotoxic T cells:	CD45$^+$CD11b$^-$B220$^-$CD3$^+$CD4$^-$CD8$^+$
Natural killer (NK) cells:	CD45$^+$CD11b$^-$B220$^+$CD3$^-$NK1.1$^+$

Figure S7. Representative gating strategy for identifying cardiac immune cell populations during infection by flow cytometry. Cells isolated from cardiac tissue were stained with a multi-parameter panel of fluorochrome-conjugated antibodies against lymphoid and myeloid surface

markers and analyzed by flow cytometry. The gating strategy began with the exclusion of debris and doublets using FSC-A/SSC-A, FSC-A/FSC-H, and SSC-A/SSC-H scatter plots, followed by the removal of non-viable cells based on viability dye uptake. Leukocytes were subsequently isolated, and myeloid and lymphoid cell subsets were resolved using defined marker combinations. Full details of the gating criteria applied to each population are provided beneath the representative plots.

Heart – schematic gating strategy

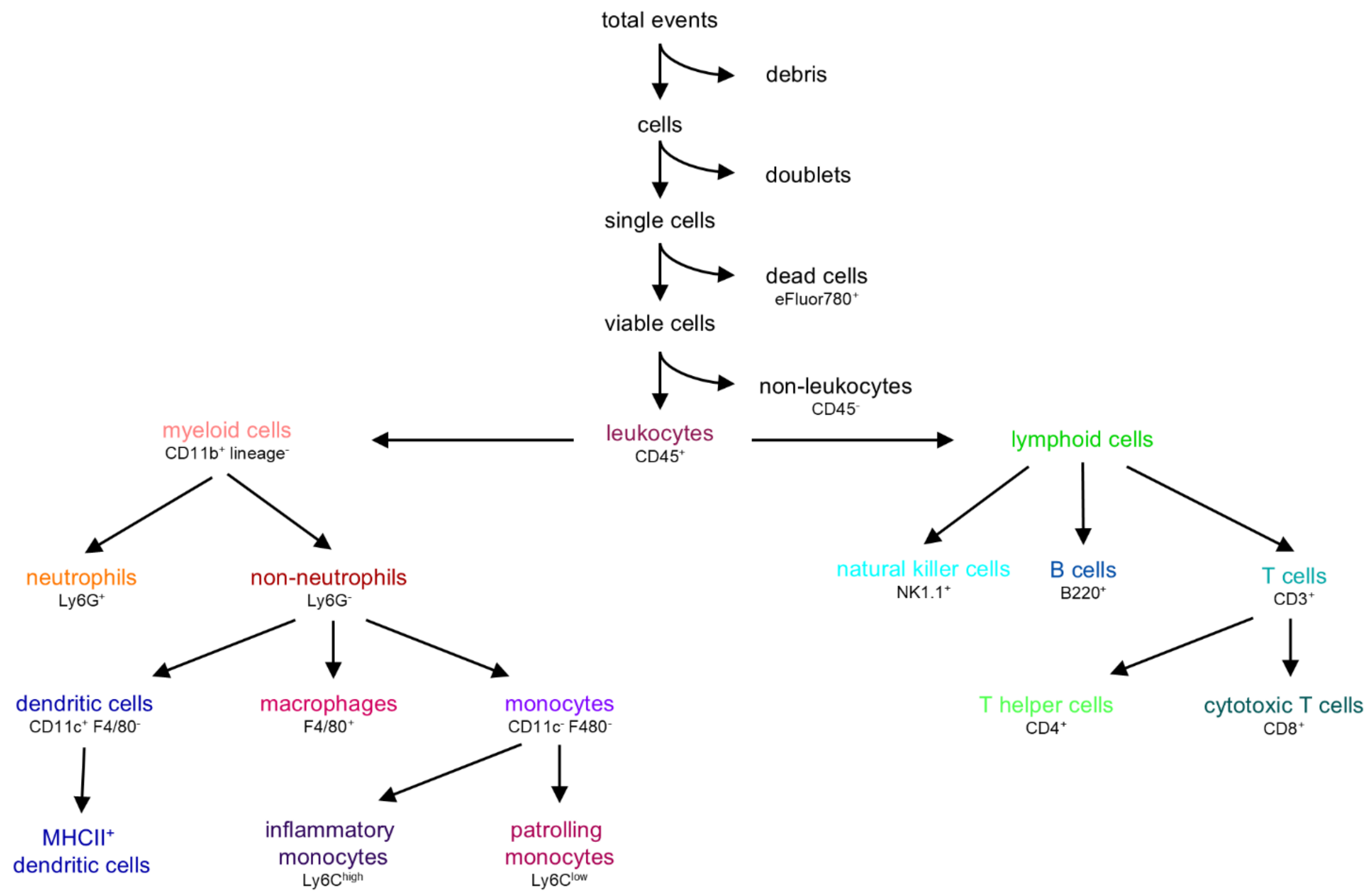

leukocytes:	$CD45^{+}$
myeloid cells:	$CD45^{+}CD11b^{+}$
neutrophils:	$CD45^{+}CD11b^{+}CD3^{-}NK1.1^{-}B220^{-}Ly6G^{+}$
macrophages:	$CD45^{+}CD11b^{+}CD3^{-}NK1.1^{-}B220^{-}Ly6G^{-}CD11c^{+/-}F4/80^{+}$
dendritic cells (DCs):	$CD45^{+}CD11b^{+}CD3^{-}NK1.1^{-}B220^{-}Ly6G^{-}CD11c^{+}F4/80^{-}$
MHCII^{+} dendritic cells (DCs):	$CD45^{+}CD11b^{+}CD3^{-}NK1.1^{-}B220^{-}Ly6G^{-}CD11c^{+}F4/80^{-}MHCII^{+}$
monocytes:	$CD45^{+}CD11b^{+}CD3^{-}NK1.1^{-}B220^{-}Ly6G^{-}CD11c^{-}F4/80^{-}$
inflammatory monocytes:	$CD45^{+}CD11b^{+}CD3^{-}NK1.1^{-}B220^{-}Ly6G^{-}CD11c^{-}F4/80^{-}Ly6C^{high}$
patrolling monocytes:	$CD45^{+}CD11b^{+}CD3^{-}NK1.1^{-}B220^{-}Ly6G^{-}CD11c^{-}F4/80^{-}Ly6C^{low}$
lymphoid cells:	$CD45^{+}CD11b^{-}$
B cells:	$CD45^{+}CD11b^{-}NK1.1^{-}B220^{+}CD3^{-}$
T cells:	$CD45^{+}CD11b^{-}B220^{-}CD3^{+}$
T helper cells:	$CD45^{+}CD11b^{-}B220^{-}CD3^{+}CD4^{+}CD8^{-}$
cytotoxic T cells:	$CD45^{+}CD11b^{-}B220^{-}CD3^{+}CD4^{-}CD8^{+}$
NK cells:	$CD45^{+}CD11b^{-}B220^{+}CD3^{-}NK1.1^{+}$

Figure S8. Schematic overview of the flow cytometry gating hierarchy for cardiac immune cell populations. This diagram depicts the sequential gating strategy employed in the flow cytometry analysis, providing a condensed summary of the representative gating plots shown in Figure S7. The specific marker combinations and gating criteria used to define each immune cell population are indicated beneath the schematic.

Table S1. Flow cytometry antibody panels for immune cell quantification. Table S1 lists all antibody-fluorochrome conjugates used for flow cytometric quantification of immune cells in heart and liver tissue, including clones, dilutions, suppliers, and catalogue numbers.

**Antibodies used for flow cytometry of heart tissue**				
antibody - fluorochrome	clone	dilution	company	catalogue number
B220 - BUV395	RA3-6B2	1:200	BD	563793
CD8 - PB	53-6.7	1:100		558106
MHCII - FITC	AF6-120.1	1:150		553551
CD3 - BUV737	145-2C11	1:200		612771
CD11b - BV510	M1/70	1:300	Biolegend	101245
NK1.1 - PE	PK136	1:300		108707
CD11c - PE dazzle	N418	1:200		117348
Ly6G - BV605	1A8	1:400		127639
F4/80 - APC	BM8	1:100		123116
CD4 - PerCPCy5.5	RM4-5	1:300		100539
CD45.2 - BV711	104	1:200		109847
Ly6C - PECy7	HK1.4	1:400		128018
**Antibodies used for flow cytometry of liver tissue (lymphoid panel)**				
antibody - fluorochrome	clone	dilution	company	catalogue number
CD8 - PB	53-6.7	1:100	BD	558106
CD4 - V500	RM4-5	1:100		560782
CD45 - BV711	104	1:300		563685
CD3 - BUV395	500A2	1:400		740221
B220 - FITC	RA3-6B2	1:200	Biolegend	103206
CD19 - PECy7	6D5	1:400		115519
NK1.1 - PE-TR	PK136	1:300		108748
CD69 - APC	H1.2F3	1:300		104513
CD62L - AF700	MEL-14	1:200		104426
CD44 - PE	IM7	1:400	Life Technologies	12-0441-81
**Antibodies used for flow cytometry of liver tissue (myeloid panel)**				
antibody - fluorochrome	clone	dilution	company	catalogue number
MHCII - FITC	AF6-120.1	1:150	BD	553551
TIM4 - BV421	21H12	1:300		742773
CD86 - BUV395	GL1	1:200		564199
CD11b - BV510	M1/70	1:300	Biolegend	101245
Ly6G - BV605	1A8	1:400		127639
CD169 - PerCPCy5.5	3D6.112	1:150		142409
CD3 - PE	145-2C11	1:300		100307
B220 - PE	RA3-6B2	1:300		103208
Ter119 - PE	TER-119	1:300		116208
CD49b - PE	DX5	1:300		108908
CD11c - PE-TR	N418	1:200		117348
Ly6C - PECy7	HK1.4	1:400		128018
F4/80 - APC	BM8	1:100		123116
CD45.2 - AF700	104	1:200		109822
